# Electrochemical Characterization of the Antioxidant Properties of Medicinal Plants and Products: A Review

**DOI:** 10.3390/molecules28052308

**Published:** 2023-03-02

**Authors:** Guzel Ziyatdinova, Alena Kalmykova

**Affiliations:** Analytical Chemistry Department, Kazan Federal University, Kremleyevskaya, 18, 420008 Kazan, Russia

**Keywords:** plants, phytotherapy, antioxidants, total antioxidant parameters, antioxidant capacity, radicals, electrochemical methods, chemically modified electrodes

## Abstract

Medicinal plants are an important source of bioactive compounds with a wide spectrum of practically useful properties. Various types of antioxidants synthesized in plants are the reasons for their application in medicine, phytotherapy, and aromatherapy. Therefore, reliable, simple, cost-effective, eco-friendly, and rapid methods for the evaluation of antioxidant properties of medicinal plants and products on their basis are required. Electrochemical methods based on electron transfer reactions are promising tools to solve this problem. Total antioxidant parameters and individual antioxidant quantification can be achieved using suitable electrochemical techniques. The analytical capabilities of constant-current coulometry, potentiometry, various types of voltammetry, and chrono methods in the evaluation of total antioxidant parameters of medicinal plants and plant-derived products are presented. The advantages and limitations of methods in comparison to each other and traditional spectroscopic methods are discussed. The possibility to use electrochemical detection of the antioxidants via reactions with oxidants or radicals (N- and O-centered) in solution, with stable radicals immobilized on the electrode surface, via oxidation of antioxidants on a suitable electrode, allows the study of various mechanisms of antioxidant actions occurring in living systems. Attention is also paid to the individual or simultaneous electrochemical determination of antioxidants in medicinal plants using chemically modified electrodes.

## 1. Introduction

Medicinal plants have been used in the treatment of various human diseases since ancient times. Their therapeutic effect is caused by the presence of a wide range of bioactive constituents with antibacterial, anti-inflammatory, antioxidant, anticarcinogenic, and antiviral properties [1,2]. Nowadays, phytotherapy and aromatherapy are mainly considered as useful supplements to basic therapy, although also applied independently in some cases. Various types of plant-material-based pharmaceutical products (extracts, decoctions, infusions, tinctures, oleoresins, and essential oils) are available for application [3]. On the other hand, medicinal plants can play a significant role in novel drug synthesis and development [4,5]. Progress in this field is caused by the development of novel methods for the identification, isolation, and purification of active principles from plant materials.

Antioxidants contained in medicinal plants are among the most actively studied constituents. The interest is caused by a diversity of antioxidants and a wide spectrum of biological activity including anti-inflammatory, anti-aging, anti-atherosclerosis, anticancer, etc. [6,7]. Antioxidants in medicinal plants are chemically variable secondary metabolites that are synthesized in various parts of the plant at different vegetation stages [8]. The phytochemical profile of medicinal plants is strongly affected by the chemotypes of plant species, the place of their origin, seasonal climate variations, environmental, gathering, and storage conditions, etc. [9,10,11]. These aspects significantly complicate the unification and standardization of medicinal plants. One of the approaches to solve this problem is the evaluation of the antioxidant properties of medicinal plants and products on their basis.

Phenolic antioxidants and terpenes as well as ascorbic acid and tocopherols are the major antioxidants of medicinal plants. The group of phenolic antioxidants (phenolic acids, flavonoids, anthocyanins, lignans, and stilbenes) is the most representative and contains more than 10,000 compounds to date [12]. Terpenes are also presented by a wide range of hydrocarbon- and oxygenated mono- and sesquiterpenes, abietane, cassane, icetexane, and neo-clerodane diterpenes, lupane, quinonemethide, ursane, and oleanane triterpenes [13,14]. Among them, phenolic monoterpenes are the major contributors to antioxidant properties.

Evaluation of the antioxidant properties of medicinal plants and pharmaceutical products on their basis is usually performed by spectrophotometry and fluorimetry. The assays are based on the reactions of antioxidants with radical species or the reduction of metal ions [15,16,17]. Typical antioxidant parameters are as follows:

- Trolox equivalence antioxidant capacity (TEAC) based on the ability of antioxidants to scavenge 2,2′-azino-bis(3-ethylbenzothiazoline-6-sulfonic acid) (ABTS^•^) radical [18,19,20,21,22,23];
- Antioxidant capacity based on the reaction of antioxidants with 2,2-diphenyl-1-picrylhydrazyl (DPPH^•^) [18,19,20,21,22,23,24];
- Oxygen radical absorbance capacity (ORAC) based on the inhibition of peroxyl-radical-induced oxidation [24,25,26,27];
- Total radical trapping antioxidant potential (TRAP) reflecting the ability of antioxidants to suppress the oxidation of 2,2′-azobis-2-methyl-propanimidamide, dihydrochloride (AAPH), or 2,2′-azobis(2-amidinopropane) dihydrochloride (ABAP) [28,29];
- Inhibition of low-density lipoprotein (LDL) oxidation based on the antioxidant effect on the peroxidation of linoleic acid or LDL induced by Cu(II) or AAPH [30,31];
- Total phenolic contents by reaction with Folin–Ciocalteu reagent based on the reduction of molybdotungsto-phosphoric heteropolyanion to molybdenum-tungsten blue [18,20,23,24,27,30];
- Ferric-reducing antioxidant power (FRAP) based on the reduction of 2,4,6-tripyridyl-S-triazine complex of Fe^3+^ in reaction with the antioxidants [18,19,22,23,32];
- Cupric reducing antioxidant capacity (CUPRAC) based on the reduction of the Cu(II)-neocuproine complex by antioxidants [23,33,34,35].

These methods have advantages and disadvantages that are well discussed in detail in [16,36,37,38]. In application to medicinal plants, the key points are the necessity to use organic solvents for antioxidant extraction, application of additional reagents or several reagents, time-consuming procedures (up to 2 h), the color of the samples leading to overlapping with the reagent absorbance band, inaccurate data for the slow reactions between antioxidants and radicals, etc.

Reactions of antioxidants that determine their biological action are based on electron transfer. Therefore, electrochemical methods are promising techniques for the investigation of the antioxidant properties of individual substances and complex samples of plant origin [12,39,40,41,42,43] as far as being simple, rapid, cost-effective, eco-friendly, and in-field applicable. Nevertheless, a lot of attention is focused on plant and plant-derived foodstuffs and beverages [40,41,42,43,44], whereas medicinal plants and products on their basis are less studied.

The current review represents the analytical capabilities of electrochemical methods in the evaluation of the antioxidant properties of medicinal plants and products on their basis. Both total antioxidant parameters assay and quantification of individual antioxidants, including their simultaneous determination, are discussed. The advantages of chemically modified electrode application are demonstrated. Electrochemical enzyme-based biosensors for the evaluation of the total phenolic content in medicinal plant extracts have recently been presented and discussed in detail in [45,46,47] and will not be considered in the current review.

## 2. Antioxidant Components of Medicinal Plants

Medicinal plants are rich in various antioxidants. As was mentioned above, the major components responsible for the antioxidant properties of medicinal plants are phenolics and terpenes of various classes. Their distribution in plants is different in location and quantity. Therefore, various parts of plants (leaves, buds, flowers, stems, roots, rhizomes, barks, etc.) are usually used for medicinal purposes. Furthermore, the type of extraction also strongly affects the chemical composition and properties of the final product. Only decoctions, infusions, tinctures, and essential oils are officially approved for application among the extracts. On the contrary, a wide range of extragents and different types of extraction are used for the characterization of plant material in terms of new compound identification [16]. In fact, this knowledge opens horizons in the creation of novel pharmaceuticals for human well-being.

The antioxidant properties of medicinal plants and typical products on their basis are mainly caused by phenolic antioxidants of various classes. The presence of reactive phenolic fragments in the structure of oxygenated monoterpenes allows them to be considered phenolic antioxidants. The most common medicinal plants and their phenolic antioxidant constituents are summarized in Table 1.

Terpenes are mainly presented in the essential oils of medicinal plants [13,14]. Their antioxidant effect is caused by the presence of double bonds and a phenolic hydroxyl group that is typical for oxygenated monoterpenes such as eugenol, thymol, and carvacrol. Both types of functional groups donate electrons or electrons and protons in reaction with N- and O-centered radical species formed during oxidative stress conditions. Nevertheless, only oxygenated monoterpenes and a limited number of other terpenes are electrochemically active and can be determined using electrochemical methods.

Carotenoids (lutein, lycopene, β-carotene, etc.) are the less common type of antioxidants in medicinal plants. They do not contribute to the antioxidant properties of decoctions, infusions, tinctures, and essential oils as insoluble in water and ethanol, which are used for the preparation of these products. However, the extraction of carotenoids with non-polar solvents or methanol with further multi-step purification is applied for the isolation of these antioxidants and further application in medicine [16].

Tocopherols and tocotrienols (α-, β- or γ-, and δ-forms) are soluble in the polar organic solvents and can be found in tinctures and ethanolic extracts obtained from the fruits and seeds of medicinal plants. Typical examples are *Capsicum annuum* L. [82] and *Echinacea purpurea* (L.) Moench [83].

Ascorbic acid is one of the most abundant antioxidants in plants. It also acts as a cofactor for numerous enzymes participating in plant metabolism and proves effective stress tolerance in abiotic conditions [84].

The antioxidant effect of medicinal plants is dose-dependent, as most low-molecular-weight antioxidants, especially phenolic ones, show prooxidant properties at high concentrations [12]. Such behavior is explained by their action mechanism, in particular, the formation of free radicals after interaction with reactive oxygen/nitrogen species and transition metals such as iron and copper. Although phenoxyl radicals are more stable compared to reactive oxygen/nitrogen species, they can cause DNA damage and mutagenesis [85]. Therefore, the control of antioxidant contents and evaluation of antioxidant properties of medicinal plant-based products are of high importance and can be achieved using electrochemical methods.

## 3. Overview of Electrochemical Methods Used for the Evaluation of Medicinal Plants Antioxidant Properties

**Figure 1:**
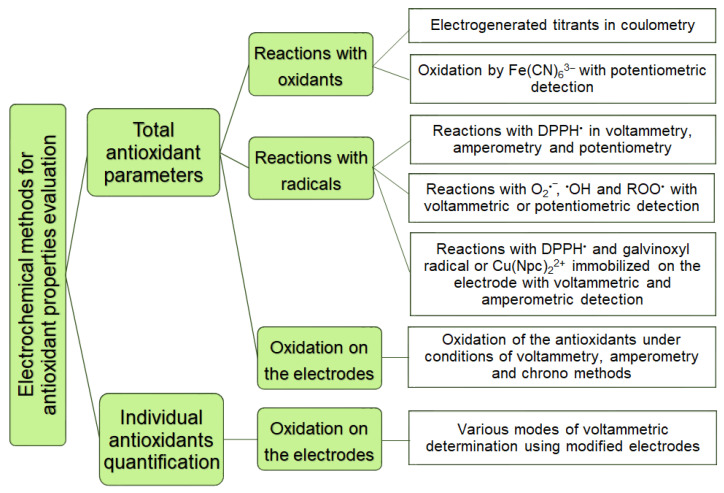
Electrochemical methods possibilities in the antioxidant properties estimation.

Various approaches to antioxidant properties estimation of medicinal plant samples can be realized using electrochemical methods (Figure 1). These include the evaluation of the total antioxidant parameters or quantification of individual antioxidants in the samples. The last one is less informative due to the multi-component composition and possible interference effect of structurally related antioxidants in the plant sample. Nevertheless, an individual determination is useful for the samples with one/two major antioxidants, whose contents are used for standardization or quality control. The analytical capabilities and limitations of electrochemical methods in the investigation of the antioxidant properties of medicinal plants are discussed below.

## 4. Electrochemical Evaluation of Total Antioxidant Parameters

Total antioxidant parameters reflect the total contents of the antioxidants in the sample and can be estimated under conditions of voltammetry, potentiometry, chrono methods, or constant-current coulometry. The measurements are based on the reactions of plant antioxidants with stable radicals, oxidants, or enzymes that are similar to the principals of spectroscopic methods. The electrochemical generation of reactive species can also be applied, which simplifies the procedure and provides more accurate data.

### 4.1. Methods Based on the Reactions with Oxidants

**Figure 2:**
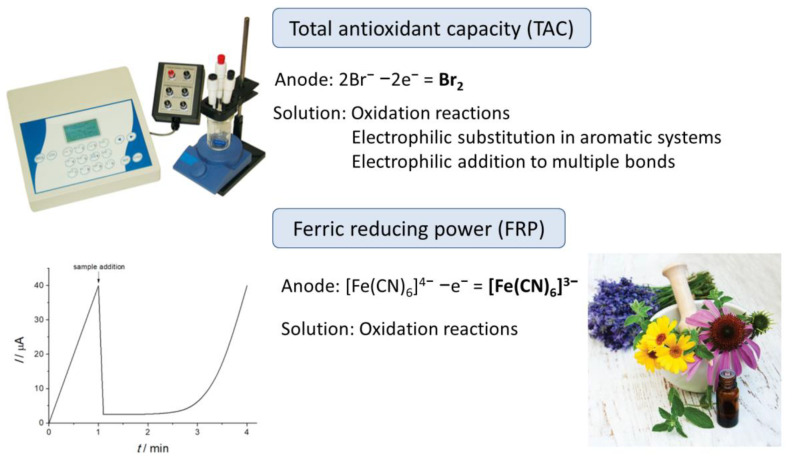
Schematic representation of coulometric methods for the evaluation of TAC and FRP.

Constant-current coulometry with electrogenerated titrants, in particular, bromine and ferricyanide ions, has been shown as an effective tool for the evaluation of total antioxidant parameters of plants and plant-derived products [42]. The method is based on the electrochemical generation of the titrant at the constant current on the platinum anode followed by a reaction with the antioxidants in the solution, which is controlled with biamperometric detection (Figure 2). Taking into account, that coulometry is an absolute method, in situ electrochemical generation of the titrants means that titration of the antioxidants with electrons takes place that excludes the necessity to use a standard antioxidant [42].

The reactions of antioxidants with electrogenerated bromine include oxidation, electrophilic addition to multiple bonds, and electrophilic substitution in the aromatic systems. Various mechanisms of antioxidant action are taken into account in this case. Therefore, coulometric titration with electrogenerated bromine allows for measuring the total antioxidant capacity (TAC) contributed by ascorbic acid, phenolic antioxidants of various classes, terpenes, and tocopherols [42]. Electrogenerated ferricyanide ions act as oxidants only, and ferric-reducing power (FRP) can be estimated. Based on the reactivity of the titrant, FRP reflects the total contents of phenolic antioxidants in the sample. Constant-current coulometry has been successfully applied for the evaluation of TAC of aqueous extracts and tinctures of 35 phytopreparations [86], *Rhodiola rosea* L. extract [87], as well as for the screening of essential oils from fifteen types of plant materials by TAC and FRP [88]. Both TAC and FRP are expressed as a quantity of electricity spent for the titration of the sample of a reasonable amount (100 g of dry plant or 100 mL for the infusions and tinctures, 1 mL for the essential oils). Simple recalculation in the equivalents of the desired standard antioxidant can be easily performed. TAC and FRP of essential oils are positively correlated (*r* = 0.7051–0.9558) with the standard spectrophotometric DPPH^•^ test and total phenolic contents by the Folin–Ciocalteu method [88]. The method is simple, highly sensitive, rapid (40 s of titration is enough to obtain reliable results with sufficient precision), uses a low volume of the samples (10–50 µL of as-prepared or diluted solutions), and can be easily automated. Furthermore, the coulometric approach has no limitations in the analysis of colored samples as has spectrophotometry. FRP reflecting total phenolic contents can be applied to any essential oil, contrary to the Folin–Ciocalteu method, for which turbid solutions are formed after the addition of reagents to a wide range of samples [88].

**Figure 3:**
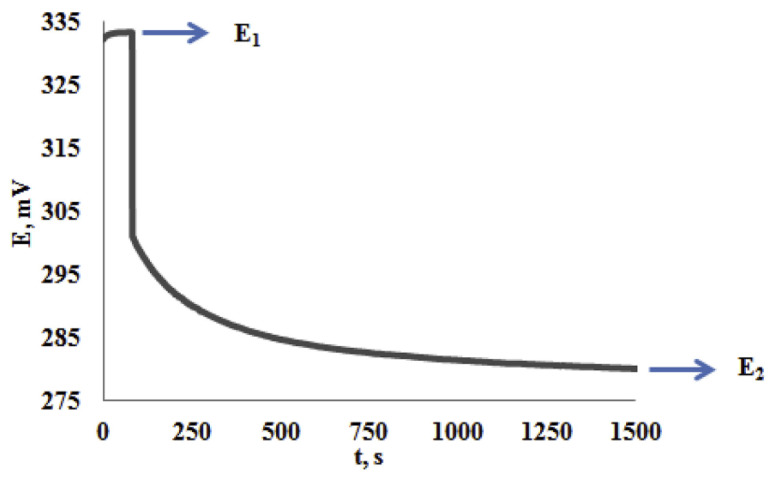
The change of the potential of K_3_[Fe(CN)_6_] (0.01 M)/K_4_[Fe(CN)_6_] (0.0001 M) system in time after the addition of 0.1 mM quercetin. Reprinted from [90], Copyright 2020, with permission from Elsevier.

The reaction of antioxidants with potassium hexacyanoferrate (III) as an oxidant has been used for the potentiometric evaluation of TAC [89,90,91]. The method is based on the change of the platinum electrode potential in the ferri/ferrocyanide system after a reaction with plant sample antioxidants (Figure 3) in phosphate buffer pH 7.4.

TAC reflects the effective equivalent concentration of antioxidants interacted with ferricyanide ions and recalculated per 1 L of the sample (Equations (1) and (2)).

(1) $$\mathrm{AOC}=\frac{c_{\mathrm{Ox}}-\alpha c_{\mathrm{Red}}}{1+\alpha }\times q,$$

(2) $$\alpha =\frac{c_{\mathrm{Ox}}}{c_{\mathrm{Red}}}\times 10^{\frac{(E_{2}-E_{1})F}{2.3RT}},$$

where *c*_Ox_ is K_3_[Fe(CN)_6_] concentration (M), *c*_Red_ is K_4_[Fe(CN)_6_] concentration (M), *E*_1_ and *E*_2_ are potential measured before and after the addition of a test sample, respectively (V), and *q* is a dilution factor (*q* = 1 for individual compounds, *q* = 15–50 for water extracts).

Natural phenolics, ascorbic acid, and α-tocopherol undergo reactions with ferricyanide ions under experimental conditions [89,90]. The major contributors to the TAC of medicinal plants are phenolic antioxidants. Infusions of 10 medicinal herbs [89], herbal teas [90], plant water extracts, and microemulsions [91] have been successfully studied and compared to standard methods. High concentrations of antioxidants (0.10–10 mM) are usually studied using a potentiometric method that is caused by its sensitivity, which can be considered as a disadvantage of the method. On the other hand, the method is very simple and easily applicable in routine practice.

### 4.2. Methods Based on the Reactions of Antioxidants with Radicals

Other electrochemical approaches are based on the application of stable radicals as reactive species which can be added to the electrochemical cell or immobilized on the electrode surface or formed electrochemically by in situ technology.

DPPH^•^ is the most widely used stable radical for the characterization of antioxidant properties. It can also be applied under conditions of electrochemistry. DPPH^•^ reduction currents are usually measured before and after the reaction with the sample antioxidants and the difference is used to calculate the TAC. Various electrochemical techniques can be applied.

**Figure 4:**
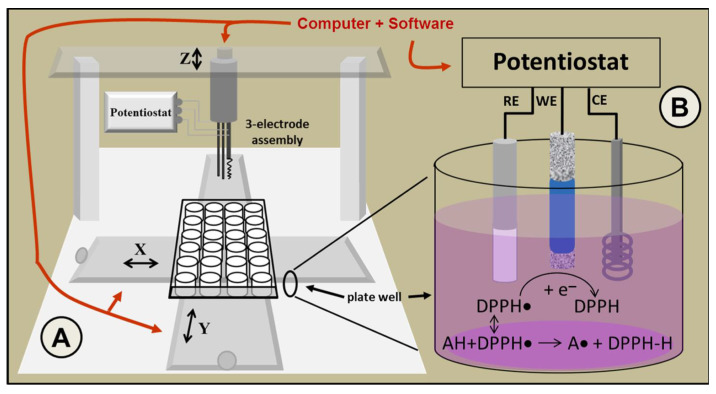
Automated DPPH^•^-based electrochemical antioxidant scaling in microtiter plates. (**A**) Coordinated x/y movements of a standard 6 × 4 well microtiter plate and z positioning of a three-electrode assembly are used to address the 24 individual wells serially for the execution of measurements and for electrode pretreatment or cleaning. (**B**) For amperometric measurements of free radical scavengers, a DPPH^•^ reduction current is established at 0.1 V and a suitable DPPH^•^ level (e.g., 100 μM). Reprinted with permission from [92]. Copyright 2012 American Chemical Society.

An original automatic amperometric approach has been developed for the screening of DPPH^•^-based TAC of Thai plant extracts [92]. It is based on 24-well microtiter plates and the three-electrode system consisted of a pencil lead working electrode, a Pt counter-electrode, and an Ag/AgCl reference electrode (Figure 4). Electrodes move sequentially through the 24 vials, containing DPPH^•^ in ethanolic phosphate buffer (pH 7.4, 0.1 M KCl), and measure reduction currents at 0.1 V before and after the reaction with the antioxidants. TAC is expressed in Trolox equivalents. The advantages of the approach include the possibility to perform analysis of a large series of samples, minimization of operator errors in measurements, and the absence of the time-consuming cleaning of electrochemical cells between measurements.

A similar way to increase the output of the determination procedure as well as the possibility of miniaturization has been presented using batch-injection analysis with amperometric detection of DPPH^•^-based TAC of plant extracts [93]. Response of a glassy-carbon electrode is selective to DPPH^•^ at 0.05 V in 0.2 M acetate buffer (pH 5.5) in ethanol (40:60, *v*/*v*). The dispensing rate value of 153 μL s^−1^ and injection volume of 80 μL have been used. The 180 injections per hour can be performed which is much more compared to conventional amperometric systems for DPPH^•^ detection [94]. The TAC has been expressed as the EC_50_ value corresponding to the concentration of the sample providing 50% inhibition of DPPH^•^.

**Figure 5:**
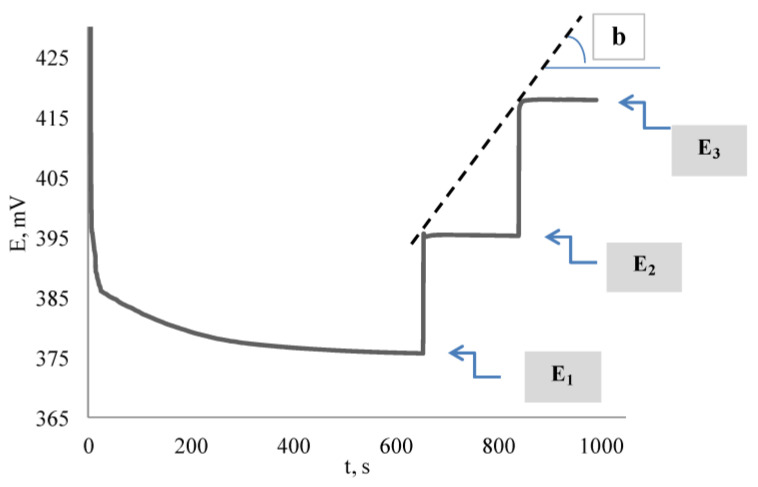
The dependence of the potential on time in introducing the 0.1 mM quercetin to the 1 mM DPPH^•^—*E*_1_; the 1 mM DPPH^•^ (c′_DPPH•_)—*E*_2_; and 3 mM DPPH^•^ (*c*′′_DPPH•_)—*E*_3_. Reprinted from [95]; published under Creative Commons CC BY license.

Recently, potentiometric monitoring of the reactions between antioxidants and stable radicals in the solution has been realized using DPPH^•^ [95]. The reaction of DPPH^•^ with antioxidants leads to the formation of DPPH-H. Therefore, the redox pair consisting of oxidized DPPH^•^ and reduced DPPH^•^-H formed after the reaction with the antioxidants is used as a potential-determining system in the potentiometric measurements. The principle of the approach is similar to the ferri/ferrocyanide system discussed above [89,90,91]. The only difference is that the Nernst slope is required to correct the evaluation of the antioxidant capacity [95]. Therefore, a sample addition to the system is followed by two consecutive DPPH^•^ additions (Figure 5). Such an approach allows the calculation of the Nernst slope by constructing a calibration curve against the background of the reaction with antioxidants.

The antioxidant capacity can be calculated by Equations (3) and (4):

(3) $${\mathrm{AOC}}_{{\mathrm{DPPH}}^{•}}=\frac{c_{{\mathrm{DPPH}}^{•}}(1-k)-kc\prime_{{\mathrm{DPPH}}^{•}}}{1-k},,$$

(4) $$k=10^{\frac{\left(E_{1}-E_{2}\right)}{b}},,$$

where *c*_DPPH•_ is the initial concentration of DPPH^•^ (M), *c*′_DPPH•_—concentration of DPPH^•^ in the first addition (M), *E*_1_—the potential of the DPPH^•^ solution, measured after the antioxidant addition (V), *E*_2_— the potential after the addition of the DPPH^•^ solution (V), and *b*—the Nernst slope. The antioxidant capacity of infusions of medicinal plants and their water–ethanol extracts (*Calendulae flores*, *Herba Leonuri*, *Folia Betulae*, *Hyperici herba*, *Herba Origanum vulgare*, *Menthae piperitae folia*, *Clitoria ternatea*, and *Hibiscus sabdariffa* L.) has been evaluated and compared to traditional spectrophotometric data [95]. The inapplicability of the last one to several samples due to intensive red and blue color has been successfully overcome using a potentiometric approach.

**Scheme 1:**
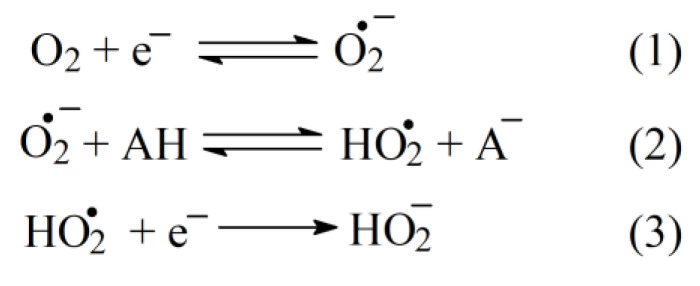
Electroreduction of oxygen with the formation of superoxide anion radical (1) and its subsequent transformation ((2) and (3)).

Among radicals, oxygen radicals are of practical interest as far as they are formed in living systems and participate in oxidative stress development [96]. The most common voltammetric approach is based on reactions of the electrochemically generated superoxide anion radical (O_2_^•–^) with antioxidants of various types (phenolics, ascorbic acid, tocopherols) [97,98,99,100,101,102,103]. Oxygen dissolved in the supporting electrolyte undergoes reversible one-electron electroreduction on the electrode with the formation of superoxide anion radical (Scheme 1 reaction (1)), which interacts with antioxidants (Scheme 1 reaction (2)).

**Figure 6:**
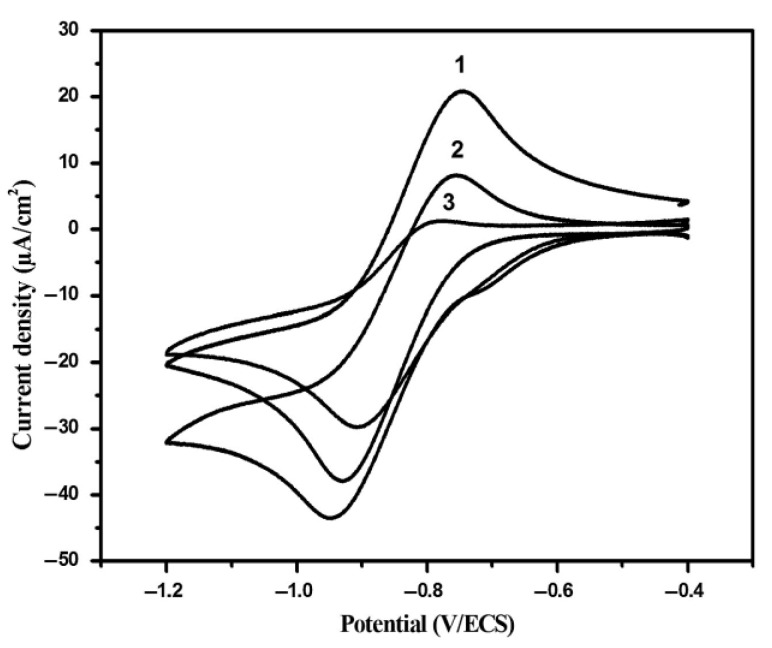
CVs of O_2_ reduction in the absence of antioxidant (1), in the presence of *Limoniastrum feei* aqueous extract (2) and ascorbic acid (3) on a steady glassy carbon disk electrode in 0.1 M Bu_4_NPF_6_ in DMF. Scan rate 50 mVs^−1^. Reprinted from [98]; published under Creative Commons CC BY license.

The reduction currents of oxygen are increased after the reaction with the antioxidants, whereas oxidation currents are decreased which is caused by reactions (2) and (3). Cyclic voltammetry (CV) is used for the reactions monitoring and typical curves are presented in Figure 6 [98].

Electrolysis is performed in the presence of a supporting electrolyte (tertiary ammonium salts) in dimethylformamide (DMF) [97,98,99,103], dimethylsulfoxide (DMSO) [100], or acetonitrile [101], which are rich in dissolved oxygen (up to mM level) using a glassy carbon (GCE) [97,98,99,100,101], or platinum [102] and multi-walled carbon nanotubes modified (MWCNTs/GCE) electrodes [103].

Superoxide scavenging capacity is usually expressed as *I*_a50_, i.e., 50% inhibition of the radicals or as an antioxidant coefficient (K_ao_) (Equation (5)) [100,101,102].

(5) $$K_{\mathrm{ao}}=\frac{\Delta I}{(I_{\mathrm{ox}}-I_{\mathrm{res}})\Delta c},$$

where Δ*I* is the change in the oxygen reduction current after adding the sample, *I*_ox_ is the limiting current of the oxygen reduction without antioxidants in the solution, *I*_res_ is the residual current without oxygen in the solution, and Δ*c* is the change in the antioxidant concentration in g mL^−1^. The conditions of the superoxide scavenging capacity measurement and plant samples studied are summarized in Table 2.

This approach is simple and rapid and does not require expensive unstable reagents. On the other hand, significant distortion of the CVs shape can occur after the addition of the plant extracts, which complicates the calculation of the superoxide scavenging capacity and makes the data unreliable. Furthermore, phenolic antioxidants of various natures change oxygen electroreduction CVs in a different way (appearance of cathodic pre-peak and/or one or more broad anodic post-peaks) depending on the structure of the phenolic compound [104,105]. Similar effects can be obtained in the case of medicinal plant samples [102].

**Scheme 2:**
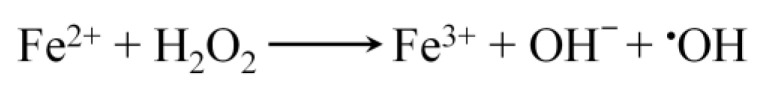
Fenton reaction.

Hydroxyl radicals are one of the most reactive and harmful among reactive oxygen species and their interaction with antioxidants is of high interest. The only reported to-date approach for the evaluation of the antioxidant effect of plant material/extracts in reactions with hydroxyl radicals is based on the exposure of the electrode in the solution where a Fenton-type reaction occurs [106,107,108]. A Fenton reaction is a way to produce hydroxyl radicals in vivo via interaction between transition metals and H_2_O_2_ (Scheme 2).

**Figure 7:**
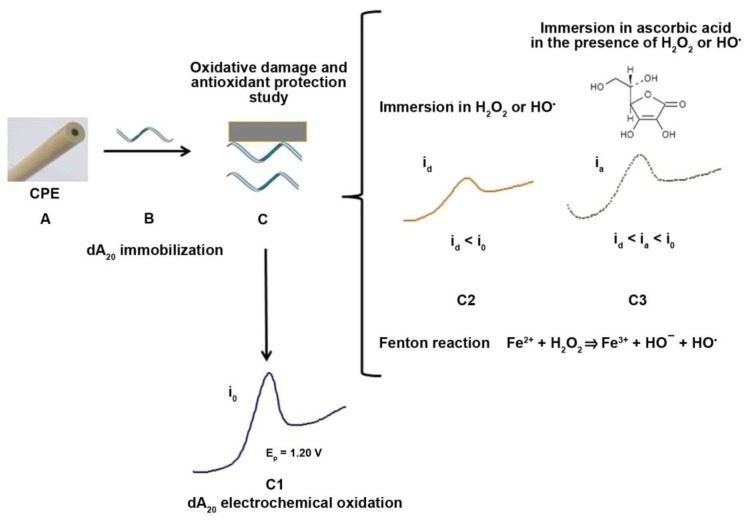
Principal scheme of DNA-based sensor design and its application in assessing hydroxyl radicals scavenging capacity. Reprinted from [107], Copyright 2016, with permission from Elsevier.

**Figure 8:**
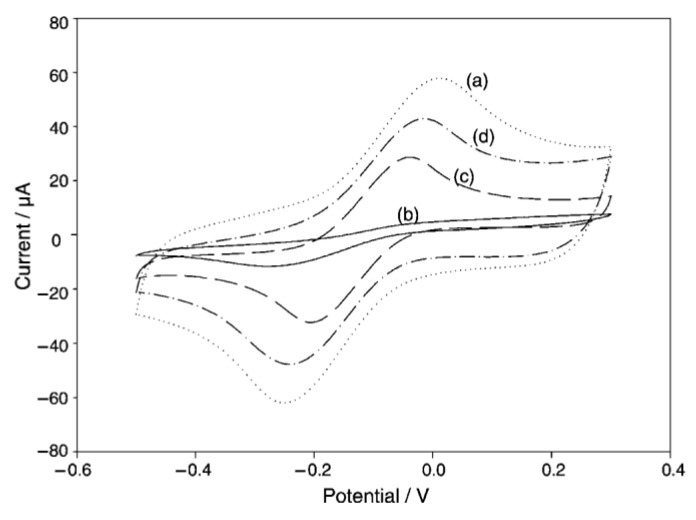
CVs of a gold electrode in a 1.0 mM [Ru(NH_3_)_6_]^3+^ in acetate buffer pH 4.6 (before and after modification of the electrode surface with hexanethiol (**curves a**,**b**), after 10 and 20 min exposure with hydroxyl radicals (**curves c**,**d**). Reprinted from [109], Copyright 2010, with permission from WILEY-VCH.

Therefore, a mixture of Fe^2+^ and H_2_O_2_ provides the formation of hydroxyl radicals. To evaluate the antioxidant properties of the sample, the electrode surface (carbon paste or screen-printed electrodes) is covered with DNA [106,107,108], or the gold electrode surface is modified with self-assembled monolayers of hexanethiol [109]. Then, the electrodes are exposed to the cleavage mixture in the absence and in the presence of antioxidants of the sample. Exposure time ranges from 5 s to 5 min. The damage of DNA bases or hexanethiol by hydroxyl radicals occurs at the electrode surface, and the corresponding electrochemical signals are registered. The changes in the oxidation currents of guanine in DNA structure [106,107] (Figure 7) or redox probe currents ([Co(phen)_3_]^3+^ adsorbed on the DNA layer [108] or 1.0 mM [Ru(NH_3_)_6_]^3+^ in solution [109]) (Figure 8) after reaction with hydroxyl radicals are used for the calculation of hydroxyl radicals scavenging capacity.

For DNA-based electrodes, the oxidation currents of guanine are significantly decreased after the reaction with hydroxyl radicals and become higher in the presence of antioxidants (Figure 7) [106,107,108]. The reduction currents of the adsorbed [Co(phen)_3_]^3+^ on the DNA layer also decreased after exposure to a cleavage medium due to a deep change in the double-stranded DNA structure, which involves DNA strand breaks and leads to a decrease in its ability to bind the [Co(phen)_3_]^3+^ [108]. In contrast, the redox currents of [Ru(NH_3_)_6_]^3+^ used as a redox probe for the gold electrode surface with self-assembled monolayers of hexanethiol are significantly increased after the reaction with hydroxyl radicals (Figure 8) that is caused by thiol-layer destruction [109]. In the presence of medicinal plant extracts, the increase in the redox probe currents is significantly less due to the inactivation of hydroxyl radicals by the antioxidants contained in the samples.

The hydroxyl radicals scavenging capacity in both cases is expressed as a relative inhibition percentage. These approaches have been successfully tested on 5% aqueous extracts of *Baccharis genstelloides*, *Peumus boldus*, *Foeniculum vulgare*, *Cymbopogom citratus*, *Camellia sinensis*, and Mentha piperita [106], nine infusions of individual and mixed plant materials [107], aqueous extracts of lemon balm, oregano, thyme, and agrimony [108], and 0.02% aqueous extracts of *Cymbopogon citratus* (DC. ex Nees) Stapf, *Psidium guajava* L., *Achyrocline satureoides* (Lam.) DC., *Matricaria chamomilla* L., and *Baccharis genistelloides* Pers. from the Rio Grande do Sul state (Brazil) [109]. The disadvantage of the electrochemical approach of indirect electrochemical evaluation of hydroxyl radicals scavenging capacity is the possibility of side reactions, that is, the interaction of Fe^2+^ with flavonoids with the formation of complexes and oxidation of the antioxidants by H_2_O_2_. To prevent a complexation reaction, ethylenediaminetetraacetic acid is added to the cleavage mixture [107,108].

**Figure 9:**
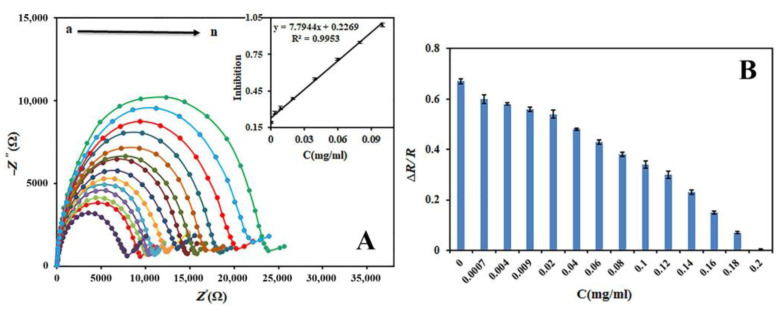
(**A**) Nyquist plots of the DNA-modified electrode after incubation with hydroxyl radicals in the absence and presence of different concentrations of the *Acanthophora* extract ((a) 0, (b) 0.0007, (c) 0.0040, (d) 0.0090, (e) 0.0200, (f) 0.0400, (g) 0.0600, (h) 0.0800, (i) 0.1000, (j) 0.1200, (k) 0.1400, (l) 0.1600, (m) 0.1800, (n) 0.2000 mg/mL); (**B**) the change of the Δ*R*/*R* values at different concentrations of the extract. Reproduced from [110], Copyright 2020, with permission from Elsevier.

An original approach for monitoring the antioxidant effect of red macro-algae *Acanthophora* extract in the prevention of DNA damage induced by the Fenton reaction has been developed using a gold nanoparticle-modified carbon screen-printed electrode with immobilized human interleukin-2 gene probe and electrochemical impedance spectroscopy detection [110]. Hydroxyl radicals have been generated in the 0.1 M Tris buffer pH 7.0 containing Cu^2+^, H_2_O_2,_ and ascorbic acid. The optimum incubation time of one hour has been found. The damage to DNA is reduced with increasing concentration of the extract achieving almost zero value at a concentration of 0.20 mg mL^−1^ (Figure 9). The increase in the semicircle diameter (charge transfer resistance *R*) on the Nyquist plot in Figure 9 indicates fewer amounts of damaged DNA as when a DNA strand break occurs, it reduces the negative charge of the DNA and leads to a decrease in charge transfer resistance [110].

**Scheme 3:**
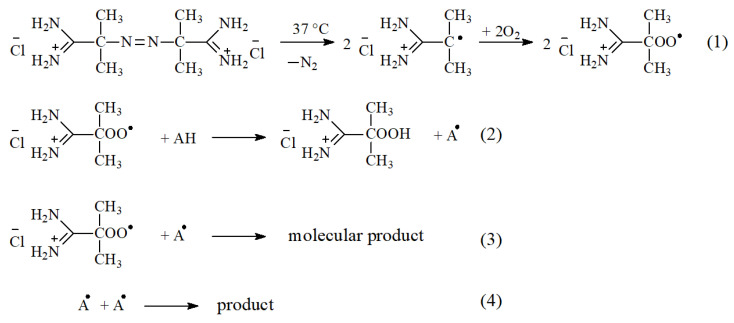
Formation of peroxyl radicals via thermal decomposition of AAPH (1) and their following reactions ((2)–(4)).

There is a lack of electrochemical methods for the evaluation of the antioxidant properties of the samples using reactions of antioxidants with peroxyl radicals. The only approach is based on the potentiometric registration of the reactions of peroxyl radicals obtained by the thermal decomposition of AAPH with natural antioxidants of plant origin [90,111]. The change of the platinum electrode potential in phosphate buffer of pH 7.4 (the total concentration of salts 0.067 M) due to the reaction of radicals with antioxidants is used as the analytical signal. The reaction of peroxyl radicals with antioxidants proceeds via several steps (Scheme 3): initiation of peroxyl radicals generation at 37 °C via decomposition of AAPH; inhibition of peroxyl radicals via reaction with antioxidants (AH in Scheme 3); loss of the antioxidants in the reactive medium [90].

**Figure 10:**
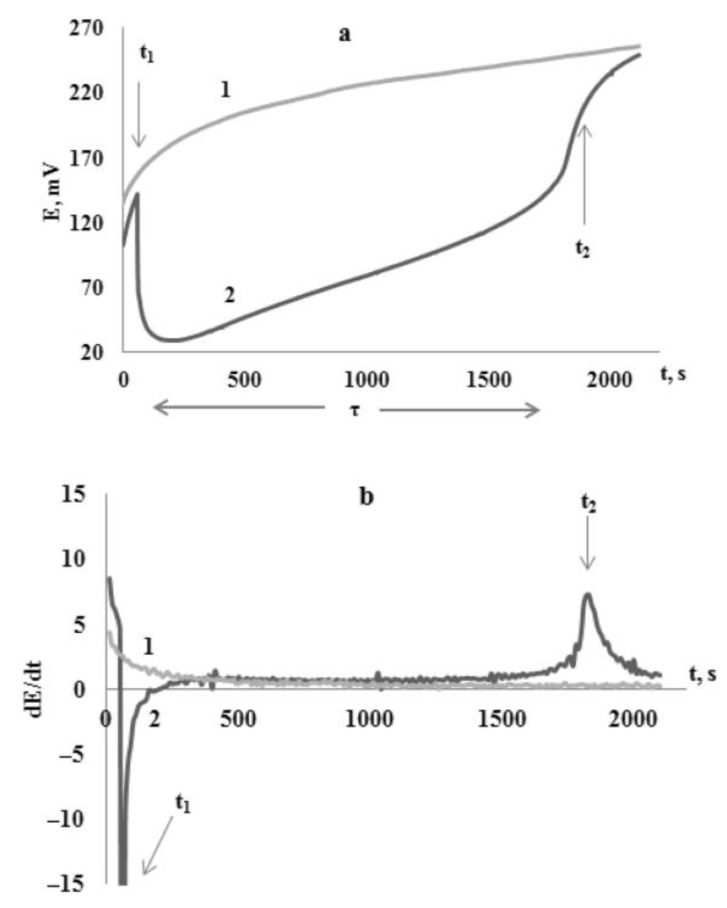
The kinetic curve of the potential change (**a**) and the derivative of the potential dependence on time (**b**): 1—0.1 M AAPH; 2—the addition of 0.2 mM ascorbic acid to 0.1 M AAPH. Reprinted from [111], Copyright 2020, with permission from Elsevier.

The initiation of the radical reaction leads to an increase in the potential in the system (Figure 10a). A significant decrease in the potential is observed after the addition of a sample containing antioxidants. The induction period (time required for the complete consumption of the antioxidants) is characterized by the stable potential value or its slight increase. Finally, the depletion of antioxidants in the system leads to a sharp increase in the potential. The differential form of the potentiometric kinetic curves (Figure 10b) has been used for the TAC calculation [111]. The approach has been successfully tested on individual antioxidants of various types and 13 commercial alcohol extracts of herbs [90,111].

### 4.3. Methods Based on the Reactions of Radicals and Oxidants Immobilized at the Electrode Surface

**Figure 11:**
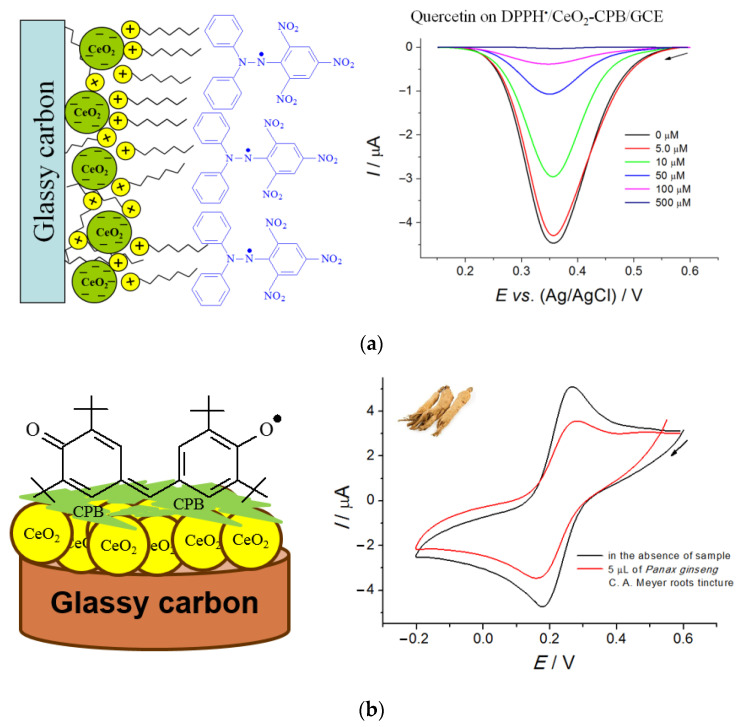
Electrodes modified with CeO_2_ nanoparticles and immobilized radicals for the evaluation of antioxidant activity of medicinal plant extracts: (**a**) DPPH^•^-modified electrode and its response. Reproduced from [112], Copyright 2017, with permission from Elsevier; (**b**) Galvinoxyl radical modified electrode and its response. Reproduced from [113], Copyright 2019, with permission from Elsevier.

Further developments in the electrochemical application of stable radicals are their immobilization on the electrode surface and voltammetric registration of their response before and after the reaction with the antioxidants. This approach has been successfully demonstrated on DPPH^•^ [112] and galvinoxyl radicals [113] immobilized on the GCE modified with CeO_2_ nanoparticles dispersed in cetylpyridinium bromide (Figure 11).

Electroreduction of both immobilized radicals proceeds reversibly with the participation of one electron. The application of electrochemically inert CeO_2_ nanoparticles provides a high surface area of the electrode for radical adsorption. The presence of cationic cetylpyridinium bromide as a dispersive agent for CeO_2_ nanoparticles and a co-modifier of the electrode surface makes possible hydrophobic interactions of DPPH^•^ or galvinoxyl aromatic rings with alkyl and aryl fragments of CPB molecules providing strong retention of radicals on the electrode surface. Differential pulse voltammetry (DPV) and CV have been used for the evaluation of the antioxidant activity of medicinal plant tinctures, decoctions, and infusions towards DPPH^•^ and galvinoxyl radical. The application of DPV in the case of DPPH^•^ is caused by the distortion of the CVs shape after reaction with the antioxidants of the sample, which makes measurement of the reduction peak current unreliable [112]. The antioxidant activity has been expressed as a relative inhibition of the radicals after 20 (for DPPH^•^) and 5 (for galvinoxyl radical) min incubation of the electrode in the phosphate buffer pH 7.4 with the addition of 5 µL of the sample.

The advantages of these voltammetric approaches are simplicity, low consumption of the reagent (2 μL instead of 3 mL), less effect of the ambient conditions such as light, oxygen, and other factors on the radicals, and the possibility to use water media of physiological pH (with negligible addition of ethanol in the case of tinctures), which does not affect the immobilized radicals compared to their solutions. The use of the galvinoxyl radical is preferable due to its high reactivity towards different types of antioxidants compared to N-centered stable radicals such as DPPH^•^. Furthermore, the electrochemical protocol for the galvinoxyl radical requires fourfold less incubation time compared to spectrophotometry (5 min vs. 20 min, respectively) [113]. The methods developed are useful for routine screening of the antioxidant activity of herbal phytopharmaceuticals.

**Figure 12:**
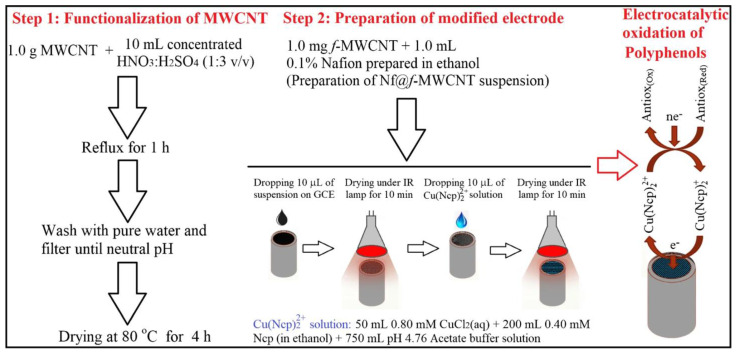
Schematic representation of preparation of the electrode modified with copper(II)-neocuproine complex and its electrocatalytic response to antioxidants (Antiox). Reprinted from [114], Copyright 2022, with permission from Springer Nature.

GCE modified with Nafion-functionalized MWCNTs and copper(II)-neocuproine complex has been used in the flow-injection analysis of TAC [114]. The schematic illustration of the electrode preparation and electrocatalytic oxidation of antioxidants is shown in Figure 12. The application of the MWCNTs-modified electrode as a platform for copper(II)-neocuproine complex allows for obtaining a very well-formed reversible redox couple for Cu(II)-/Cu(I)-complex. The increase in the anodic peak current of the redox couple in the presence of phenolic compounds such as trolox, catechin, and quercetin confirms the electrocatalytic effect towards the oxidation of these analytes.

**Scheme 4:**
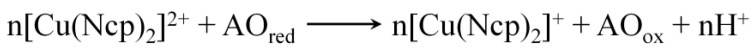
Reaction of copper(II)-neocuproine complex with antioxidants.

The determination is based on the electron transfer reaction between copper(II)-neocuproine complex ([Cu(Ncp)_2_^2+^]) and antioxidants (AO) according to Scheme 4.

Amperometric detection has been performed at +0.6 V in Britton–Robinson buffer pH 7.0 containing 0.1 M KCl. Herbal tea extracts (green tea, linden, thyme, bitter melon, and rosemary) have been successfully studied and comparable results with the spectrophotometric CUPRAC method have been obtained. This approach provides rapid, selective, low-cost, simple, portable, and direct detection of antioxidant compounds (giving higher rank to fast-reacting antioxidants) as well as higher sensitivity and sample throughput than traditional spectrophotometric and voltammetric methods.

### 4.4. Methods Based on the Oxidation of Antioxidants on Electrodes

Another approach to evaluate the antioxidant contents in medicinal plant samples is their oxidation on the surface of suitable electrodes under conditions of voltammetry or chrono methods. In this case, the results are more reliable as far as no necessity to use additional reagents which are affected by environmental factors, measurement, and storage conditions. The reproducibility of electrooxidation is also higher than for the chemical reaction with radical species.

**Figure 13:**
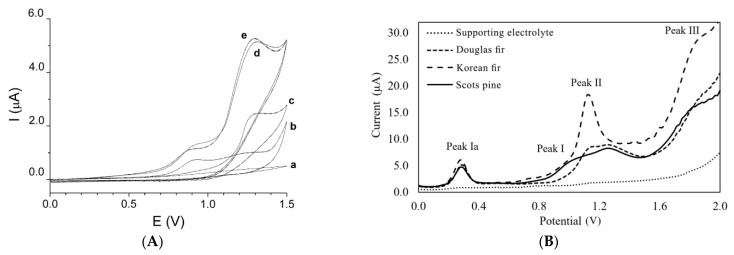
(**A**) CVs of 4.0 mg mL^−1^ *Bunchosia glandulifera* tree extract from fruit pulp (curve (a)), seed (b), bark (c), leaf (d), and root (e) on the GCE in 0.1 M Bu_4_NPF_6_ in DMSO. Reproduced from [116], Copyright 2017, with permission from Elsevier; (**B**) DPV electrooxidation of cone extracts in 0.1 M Bu_4_ NClO_4_ in acetonitrile on the Pt electrode. Reproduced from [117]; published under Creative Commons CC BY license.

Various types of voltammetry (CV, DPV, or square-wave (SWV)) are shown to be effective tools for the evaluation of antioxidant capacity. CV is a relatively seldom applied technique in the quantitative analysis of medicinal plants due to insufficient sensitivity. On the other hand, the area under the voltammetric curve or oxidation peak gives the quantity of electricity and can be used for the characterization of total antioxidant contents using total charge [115,116]. Two oxidation peaks have been observed at 0.9 and 1.3 V on the GCE for the root, leaf, and seed extracts of the *Bunchosia glandulifera* tree, whereas the only peak at 1.3 V for the bark extract and no peaks for the fruit pulp extract have been obtained [116] (Figure 13A). Such behavior of the extracts is caused by types of phenolics and can be used for TAC determination. Ascorbic acid has been chosen as a standard that is not entirely correct as far as the oxidation peaks are caused by the presence of phenolic antioxidants (flavonoids and phenolic acids). Therefore, gallic acid or quercetin could be a more appropriate standard to express the total antioxidant parameters, as they are presented most frequently in plant samples and give a stable response in all methods used to date.

Methanolic extracts of seven medicinal plants (*Buxus hyrcana, Rumex crispus, Achillea millefolium*, *Zataria multiflora*, *Ginkgo biloba, Lippia citriodora*, and *Heptaptera anisoptera*) have been studied by CV using oxidation potential and currents data [118]. The presence of oxidation peaks at around +0.3 V on GCE on the voltammograms has been considered as a strong free radical scavenging capability of the extracts. The oxidation current values reflect antioxidant concentration in the sample. Thus, *Rumex crispus*, *Achillea millefolium,* and *Ginkgo biloba* showed higher antioxidant capacity than the others, whereas *Lippia citriodora* and *Zataria Multiflora* contained higher amounts of antioxidants. This approach is valid for the comparison of extracts prepared by the identical protocol and the same ratio of raw material to extragent. The incorrect interpretation of the data can occur in the case of infusions, decoctions, and extracts used in phytotherapy and medicine because each of these products is prepared from various amounts and types of plant materials taking into account their properties (hardness, porosity, wettability, etc.) and pharmacological effects.

DPV and SWV are more sensitive compared to CV and have been used more often for the characterization of medicinal plant extracts. Similarly to CV, oxidation peaks are registered on the voltammograms (Figure 13B) [117]. The oxidation potentials are reflected by the antioxidant constituents, and the oxidation currents depend on the concentration of the antioxidants. Based on the oxidation potentials of the sample, A. Escarpa et al. have suggested the parameter “electrochemical index (EI)” for the determination of total polyphenols in foodstuffs using DPV [119]. The idea is based on the interpretation of the main voltammetric characteristics, i.e., the oxidation peak potential (*E*_pa_) and current (*I*_pa_). The lower *E*_pa_ (thermodynamic parameter) characterizes the higher electron donor ability, and the higher *I*_pa_ (kinetic parameter) means the higher quantity of electroactive species. EI is calculated using Equation (6):

(6) $$\mathrm{EI}=\frac{I_{p}{{}_{a}}_{{}_{1}}}{E_{\mathrm{pa}}{}_{{}_{1}}}+\frac{I_{\mathrm{pa}}{}_{{}_{2}}}{E_{\mathrm{pa}}{}_{{}_{2}}}+\frac{I_{p}{{}_{a}}_{{}_{3}}}{E_{\mathrm{pa}}{}_{{}_{3}}}+\ldots +\frac{I_{\mathrm{pa}}{}_{{}_{n}}}{E_{\mathrm{pa}}{}_{{}_{n}}},$$

where *I*_pa_ is the anodic peak current and *E*_pa_ is the potential of the same peak [119]. According to the oxidation potential value in a neutral medium (pH 7.5), phenolic antioxidants are classified as fractions of high (*E*_pa_ = +0.3 V) and intermediate (*E*_pa_ = +0.5 V) antioxidant power. The amperometric response at 0.8 V provides total phenolic content or EI. Later, EI has been used successfully in medicinal plants. Table 3 summarizes the corresponding data on DPV and SWV capabilities in the characterization of medicinal plants’ antioxidant properties.

Taking into account the complex matrix of plant samples, the area under the voltammetric curves is a more suitable and reliable parameter than the peak current due to the integral nature of the voltammetric signal caused by several antioxidants with slightly different oxidation potentials. Natural phenolic antioxidants are the main contributors to the antioxidant properties of medicinal plant extracts. Therefore, voltammetric data provide an opportunity to measure total phenolic contents. However, the minor impact of other antioxidants such as ascorbic acid or tocopherols can not be fully excluded. Therefore, TAC or its variations are a more appropriate term. Positive correlations with the standard spectrophotometric assays of total phenolics and TAC towards DPPH^•^ confirm the accuracy of the data obtained by DPV and SWV and their applicability to the medicinal plants’ analysis.

Amperometric detection of antioxidant oxidation in combination with flow-injection analysis is another approach to the evaluation of medicinal plants’ antioxidant properties [126]. Preliminary investigations of various classes of phenolic antioxidants under conditions of hydrodynamic voltammetry on GCE have shown that the applied potential of +0.5 V provides selective discrimination of compounds with high reducing capacity, hence with effective antioxidant activity. The methanolic extracts of *Rosmarinus officinalis* L., *Salvia officinalis* L., *Thymus vulgaris* L., *Origanum vulgaris* L., *Ocinum basilicum* L., *Mentha piperita*, and *Laurus nobilis* L. have been studied. Antioxidant activity has been expressed in a Trolox equivalent that simplifies the comparison of the data obtained with those reported earlier. Another advantage is the rapidity of the analysis (60 determination h^−1^) making the method attractive for routine screening purposes.

Chrono methods (chronoamperometry and chronocoulometry) did not receive enough attention in the investigation of medicinal plants and products on their basis, although it is simpler and easier in use and data interpretation compared to voltammetry. The preliminary voltammetric behavior of the plant sample is studied in order to make a choice of the potential at which the electrolysis is to be performed. One- and multi-step electrolysis allows the differentiation of various classes of antioxidants on the basis of their oxidation potentials.

**Figure 14:**
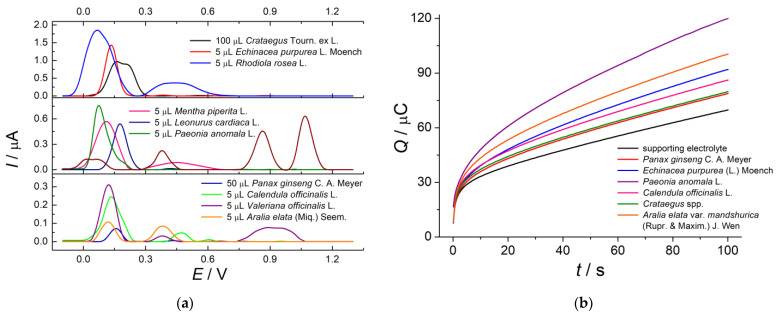
Evaluation of the medicinal plant tinctures’ antioxidant capacity: (**a**) Baseline-corrected DPVs of tinctures on poly(gallic acid)/MWCNTs/GCE in phosphate buffer pH 7.4; (**b**) Typical chronocoulometric curves of 20 μL tinctures at 1.0 V. Reproduced from [127] with permission from the Royal Society of Chemistry.

Chronocoulometry on the GCE modified with MWCNTs and electropolymerized gallic acid in phosphate buffer pH 7.4 has been developed for the evaluation of the antioxidant capacity of medicinal plant tinctures [127]. Eleven medicinal plant tinctures have been studied and all of them show oxidation peaks in the range of 0–0.3 V, the majority of the tinctures also give peaks at 0.3–0.6 V, and only two tinctures (*Valeriana officinalis* L. and *Schisandra chinensis* (Turcz.) Baill.) also show peaks at 0.75–1.2 V (Figure 14a).

Therefore, one-step chronocoulometry at 1.0 V for 100 s (Figure 14b) has been used to characterize the antioxidant capacity of the tinctures using quercetin as a standard. A strong positive correlation of data obtained and total phenolics by Folin–Ciocalteu method and antioxidant activity towards DPPH^•^ confirm the applicability of chronocoulometry in assessing the antioxidant capacity of medicinal plant tinctures and other extracts [127].

A similar approach has been tested on commercial essential oils from 15 types of plants [128]. Essential oils show oxidation peaks at 0.0–0.75 and 0.75–1.5 V on the GCE modified with carboxylated MWCNTs in phosphate buffer pH 7.0. Oxidation of phenolic antioxidants occurs in the first potential window, whereas the terpenoids are oxidized in the second range as confirmed by voltammetric characteristics of individual antioxidants and terpenoids. Thus, two-step chronoamperometry at 0.80 and 1.4 V has been used for the evaluation of the essential oils’ antioxidant capacity. The electrolysis steady-state has been achieved at 75 s for each step. The antioxidant capacity has been expressed as an oxidation current at 0.8 V reflecting the contents of phenolic antioxidants and the total antioxidant contents using a current at 1.4 V. Both parameters have been recalculated per 1 mL of essential oil. The antioxidant capacity at 0.8 V correlates with total phenolic contents by the Folin–Ciocalteu method and the TAC at 1.4 V—with DPPH^•^ test data. The chronoamperometric approach overcomes the limitations of the Folin–Ciocalteu method that is applicable to clove, cinnamon, nutmeg, and thyme essential oils only (the turbid solutions are formed for other essential oils) as well as a less tedious procedure compared to spectrophotometric methods and does not require additional reagents.

As was mentioned above, electrochemical methods are a good alternative to traditional methods such as chromatography and spectrophotometry in the evaluation of plants’ antioxidant parameters. Nevertheless, they also have advantages and disadvantages (Table 4) to be taken into account during the choice of method.

Knowledge of the major class of antioxidants in medicinal plants allows electrochemical determination of total content. Such an approach is well-developed for application to food analysis. In the case of medicinal plants, limited data have been reported. A simple, rapid, and cheap method has been developed to determine total phenolic monoterpenes in particular isopropylmethylphenols (thymol and carvacrol) in essential oils of thyme and oregano using linear sweep voltammetry on GCE in 0.1 M Bu_4_NClO_4_ in acetonitrile [129]. Being isomers, both thymol and carvacrol are usually presented in plant samples. Their oxidation proceeds at the same potential (+1.29 and +1.28 V, respectively) making their total quantification possible, which reflects the antioxidant properties of thyme and oregano since thymol and carvacrol are the main constituents. The linear dynamic ranges of 85–1300 and 79–1200 µM for thymol and carvacrol, respectively, have been achieved. Nevertheless, the sensitivity of the electrode response is not impressive and linear ranges cover high concentrations of the analytes. Furthermore, the necessity to use organic solvent and corresponding electrolyte salt increases the costs and is not eco-friendly.

Another typical example is the determination of total capsaicinoids in *Capsicum annuum* L. tinctures using GCE modified with MWCNTs and electropolymerized gallic acid [130], or GCE with carboxylated single-walled carbon nanotubes and dispersed in cetylpyridium bromide CeO_2_ nanoparticles [131]. Electrode surface modification provides an improvement in the capsaicinoids response due to the adsorption of the analytes at the electrode surface via hydrophobic interactions with polymeric coverage [130] or cetylpyridium bromide [131]. Furthermore, an increase in the effective surface area of the electrodes and electron transfer rate has been provided due to the presence of a modifier. The selectivity of the electrodes towards capsaicinoids in the presence of α-tocopherol and ascorbic acid allows measuring total capsaicinoid contents reflecting antioxidant properties of the *Capsicum annuum* L. There are well-defined anodic peaks at 0.60–0.63 V in Britton–Robinson buffer pH 2.0 on both electrodes. Two electron oxidation occurs with the formation of the *o*-quinone moiety in the structure of capsaicinoids [130,131]. Capsaicin as the major constituent has been used as a standard among the capsaicinoids. Poly(gallic acid)-modified electrode allows quantification of capsaicin in the ranges of 0.010–1.0 and 1.0–50 μM with the limit of detection of 2.9 nM [130]. The linear dynamic ranges of 0.10–7.5 and 7.5–500 μM of capsaicin with the limits of detection of 28 nM have been achieved on the electrode with carboxylated single-walled carbon nanotubes and CeO_2_ nanoparticles [131]. These analytical characteristics confirm the high sensitivity of the methods developed. The total contents of capsaicinoids found by voltammetry agree well with the chromatography [130] and spectroscopy [131] data. The main advantages of the voltammetric approaches are rapidity in comparison to chromatography, the possibility of miniaturization, and the fabrication of the single-used electrodes using screen-printed technology.

An original approach to selective detection and determination of catechins (flavan-3-ols) in the presence of other flavonoids has been developed using DPV on GCE [132]. Voltammetric measurements are performed in the presence of AlCl_3_ which forms stable complexes with flavonoids such as quercetin and rutin. Complexation leads to changes in the flavonoids’ voltammetric behavior and the disappearance of their interference effect due to the anodic shift of the oxidation potentials. The simple addition of AlCl_3_ to the supporting electrolyte allows the elimination of the interference effect of other flavonoids. The practical applicability of the method has been shown in herbal medicines.

Thus, electrochemical methods can be used successfully for the estimation of total antioxidant parameters. In some cases, the first steps in phytochemical profiling based on the fingerprints approach can be achieved using DPV or SWV including solid-state electrochemistry based on the voltammetry of immobilized microparticles of plants or their extracts [133,134,135].

## 5. Electrochemical Determination of Individual Antioxidants in Medicinal Plants

Determination of major individual antioxidants or several of them is another approach for the characterization of medicinal plants’ antioxidant properties. In this case, the major antioxidant content in the sample is considered as reflecting the antioxidant potential of the entire sample. Furthermore, this information is useful for the isolation of antioxidants that are further used in the production of pharmaceutical dosage forms and phytopharmaceuticals. A simultaneous determination of structurally related antioxidants is also required. Therefore, the selectivity of the target antioxidant response plays a crucial role since compounds of similar structure are usually presented in the sample. This problem has a great impact in relation to electrochemical methods and is one of the main limitations of their practical application in plant analysis. There are two strategies to solve this problem:

- The creation of highly sensitive electrodes to the target antioxidant, which is a major and typical component of the medicinal plant. The determination should be performed by voltammetry after a significant dilution of the sample. In this case, other antioxidants will not give a sufficient response due to the low concentration, and the signal of the target antioxidant will be enough for quantification;
- Fabrication of highly selective electrodes allowing simultaneous determination of structurally related antioxidants.

The combination of these two trends is desirable and has been realized through the development of chemically modified electrodes. The choice of a sufficient modifier can provide excellent sensitivity and selectivity of the target antioxidant determination. Electrode surface modification can be performed by the following methods [136]:

- irreversible adsorption of modifier on the electrode surface;
- chemical binding of modifier via various groups (spacers, linkers) with covalent bond formation;
- inclusion in the polymer film;
- addition in the volume of the carbon paste or composite material mechanically or using screen printing technology;
- formation of modifier layer using the sol-gel technology;
- drop casting or electrochemical formation of polymer or cavity-containing material capable to work on the guest–host principles (molecularly imprinted materials).

Various types of modifiers, in particular carbon nanomaterials, nanoparticles, and nanostructures of metal, metal oxide, sulfides, complexes and other compounds, metal-organic frameworks, ionic liquids, surfactants, and polymeric materials, have been shown to be effective electrode surface modifiers for antioxidants electroanalysis [12,137,138,139,140,141,142]. The determination of individual antioxidants in medicinal plants and products on their basis can also be achieved using chemically modified electrodes.

Flavonoids and phenolic acids are the most studied analytes, although ascorbic acid and eugenol are also of interest [143,144,145,146,147,148,149,150,151,152,153,154,155,156,157,158,159,160,161,162,163,164,165,166,167,168,169,170,171,172,173,174,175,176,177,178,179,180,181,182,183,184,185,186]. Anethole is almost out of the investigation [187], although it is often used in plant-based pharmaceuticals as a flavoring component. The analytical capabilities and application area of the electrochemical methods in the individual antioxidants quantification in medicinal plants are presented in Table 5. Raw plant materials, their extracts, and plant-based pharmaceutical dosage forms are studied.

Various types of voltammetry have been used for quantification purposes. Most approaches are based on the application of pulse modes (DPV and SWV) due to the higher sensitivity compared to the linear sweep mode. Furthermore, adsorptive preconcentration at the open circuit potential [149,150,160,168,170,177,178,179] allows for improvement of the analytical characteristics of the antioxidants quantification, although it significantly increases the measurement duration (up to 5 min) and can lead to co-adsorption of other components of the sample and complicate the determination.

Electrode fouling often occurs due to the complex composition of the plant sample that requires renewal of the electrode surface after each measurement [145,147,151,156,157,173,185]. Single-use electrodes, in particular screen-printed ones, are a good alternative to electrode cleaning after each measurement. Moreover, screen-printed electrodes with stable modifier layers such as carbon nanomaterials can be used as a platform for further deposition of sensitive layers giving a response to the target antioxidant.

As one can see, the combination of several modifiers provides better analytical characteristics of antioxidants due to the synergetic effect of several modifiers. For example, carbon nanomaterials and electrochemically inert metal oxide nanoparticles (CeO_2_, ZrO_2_, Ta_2_O_5_, La_2_O_3_, etc.) provide a high surface area of the electrode and conductivity. Electropolymerized coverages of triphenylmethane dyes and gallic acid act as selective components giving a response to phenolic antioxidants due to the structural similarity [147,151,173]. Metal and bimetal nanoparticles, and metal complexes act on the principle of electrocatalysis and provide quantification of low concentrations of antioxidants [154,165,168,178]. The combination of electrochemically inert metal oxide nanoparticles with surfactants as dispersive agents leads to the formation of stable dispersions with a smaller size of nanoparticles [145,152,186]. After deposition on the electrode surface, a significant improvement in the electrode conductivity and effective surface area has been observed. Furthermore, the possibility of selective preconcentration of the antioxidants at the electrode surface due to their hydrophobic and/or electrostatic interactions with surfactants acting as electrode co-modifier [145,152,156,162,185].

**Table 5:** Figures of merit of electrochemical methods for the individual quantification of antioxidants in medicinal plants and products on their basis.

Antioxidant	Method	Electrode	Limit of Detection (µM)	Linear Dynamic Range (µM)	Plant Sample	Refs.
Quercetin	DPV	Three-dimensional reduced graphene oxide aerogel/Carbon ionic liquid electrode	0.065	0.1–100	Ginkgo tablets	[143]
	LSV ^1^	Lewatit FO36 Nanoresin/MWCNTs/GPE ^2^	0.213	1.8–25; 25–570	Gincora tablets	[144]
	DPV	CeO_2_ nanoparticles–Sodium dodecyl sulfate/GCE	0.0029	0.010–1.00; 1.00–250	*Hyperici herba, Calendulae officinalis flores, Arctostaphyli uvae ursi folia* extracts, infusions, decoctions, hydrolysates	[145]
	DPV	Au-Co nanoparticles-embedded N-doped carbon nanotube hollow polyhedron/GCE	0.023	0.050–35	Ginkgo tablets	[146]
	DPV	Poly(gallic acid)/ MWCNTs/GCE	0.054	0.075–25; 25–100	*Arctostaphylos uva-ursi* (L.) Spreng. Leaves and *Calendula officinalis* L. flowers decoctions, infusions, and tincture	[147]
	DPV	Pt-Au alloy–Biomass-derived porous carbon nanocomposite/Carbon ionic liquid electrode	0.050	0.15–6.0; 10–25	Ginkgo tablets	[148]
Rutin	AdASWV ^3^	Mesoporous carbon and 1-butyl-3-methylimidazolium hexafluorophosphate based paste electrode	0.00117	0.008–4.0	*Ruta graveolens* extract	[149]
	AdASWV	Neodymium(III) oxide-SWCNTs ^4^ in chitosan/GCE	0.092	0.99–8.00	Extract of *Zamia furfuracea* L.f. ex Aiton	[150]
	DPV	CeO_2_ nanoparticles–Sodium dodecyl sulfate/GCE	0.028	0.10–100	*Hyperici herba* extract, infusion, decoction	[145]
Quercetin	DPV	Polythymolphthalein/Carbon nanofibers/GCE	0.00073	0.025–1.00	Infusion of *Tilia* L. flowers	[151]
Rutin			0.0047	0.025–1.00		
Quercetin	DPV	Fe_3_O_4_@ZnO core/shell magnetic nanoparticles-CPE	0.14	0.29–64.7	Borage, chamomile, asparagus, teucrium, tarragon, pennyroyal extracts	[152]
Rutin			0.07	0.099–99		
Morin	DPV	Poly(2,5-dimercapto-1,3,4-thiadiazole)/CFPE ^5^	8.3 × 10^−5^	2.5 × 10^−4^–2.75 × 10^−3^	Mulberry leaves	[153]
		Nickel (II) phthalocyanine–CPE ^6^	0.0020	0.10–2500	*Psidium guajava* leaf extract	[154]
		Gum arabic stabilized Ag nanoparticles–CPE	0.216 × 10^−3^	(0.65–7.0) × 10^−3^	Mulberry leaves	[155]
		Cetylpyridinium bromide/Carboxylated SWNTs/GCE	0.0289	0.1–100; 100–750	Mulberry leaves	[156]
Hesperidin	DPV	Nano-graphene-platelets–Brilliant green/CPE	0.050	0.1–7.0; 7.0–100.0	Peppermint extract	[157]
Dihydromyricetin	DPV	Double-layered membranes from Au nanoparticles anchored on reduced graphene oxide and polyacrylamide-based MIP ^7^/GCE	0.012	0.020–100	*Ampelopsis grossedentata* leaves extract	[158]
Naringenin	LSV	SWNTs/GCE	0.020	0.080–5.0; 5.0–12	*Fructus Aurantii* Immaturus	[159]
Hyperin	AdDPV	Poly(diallyldimethylammonium chloride)-functionalized graphene/GCE	0.005	0.007–0.70	*Hypericum Perforatum*	[160]
	DPV	α-Fe_2_O_3_ doped graphene/GCE	0.5 × 10^−3^	0.001–0.10	*Abelmoschus manihot, Semen cuscutae,* and *Chinese Herba Hypericum perforatum*	[161]
	DPV	ZrO_2_ nanoparticles–Sodium dodecyl sulfate–Carboxylated SWCNTs/GCE	0.5 × 10^−3^	0.001–0.30	*Abelmoschus manihot*	[162]
Esculetin	DPV	TiO_2_ nanoparticles-coated poly(diallyldimethylammonium chloride)-functionalized graphene/GCE	0.004	0.010–3.5	*Viola yedoensis* Makino and *Cotex fraxini*	[163]
		*sp*-Hybridized nitrogen atom doped ultrathin graphdiyne/carbon ionic liquid electrode	0.0023	0.02–10.0	Capsules, pills, and *Cortex fraxini*	[164]
Mangiferin	DPV	Au-Ag nanoparticles/MWCNTs–sulfonated graphene sheets/GCE	0.017	0.05–500.0	*Rhizoma Anemarrhenae*, *Artemisia Capillaris Herba,* and the leaves of *Epimedium Macranthum*	[165]
Iicariin			0.017	0.05–100.0		
Chrysin	LSV	Ta_2_O_5_ particles–Chitosan–CPE	0.03	0.08–4	*Oroxylum indicum*	[166]
Baicalein			0.05	0.08–4		
Baicalein	DPV	Ta_2_O_5_-Nb_2_O_5_@Chitosan–CPE	0.05	0.08–8	Scutellariae Radix	[167]
Baicalin			0.03	0.08–8		
Gallic acid	AdADPV ^8^	Pt nanoparticle–poly(diallyldimethylammonium chloride)-functionalized graphene/GCE	0.007	0.030–1.0	Jianmin Yanhou tablets, Cortex moutan	[168]
	LSV	Electropolymerized methylene blue on graphene oxide framework/GCE	49	50–1000	Herbal tablets containing *Euphorbia prostrata*	[169]
Ferulic acid	AdADPV	Electrochemically reduced graphene oxide/GCE	0.0206	0.0849–38.9	*Angelica sinensis*	[170]
	CV	Graphene oxide–MWCNTs/GCE	0.08	0.24–1230	*Pinellia ternata*	[171]
	DPV	Poly(phenol)-based MIP/Au nanoparticles/Electrochemically reduced graphene oxide/SPEa ^9^	0.0031	0.010–1.0	Orange peel	[172]
Ferulic acid	DPV	Poly(bromocresol purple)/Polyaminobenzene sulfonic acid functionalized SWCNTs/GCE	0.072	0.10–5.0; 5.0–25	*Vanilla planifolia* (two-fold and three-fold strength) extracts	[173]
Vanillin			0.064	0.10–5.0; 5.0–25		
Rosmarinic Acid	DPV	Fe_3_O_4_@SiO_2_@NH_2_ decorated poly(methacrylic acid)-based MIP–CPE	0.085	0.1–100; 100–500	*Salvia officinalis, Zataria multiflora, Mentha longifolia, Rosmarinus officinalis*	[174]
Rosmarinic Acid	DPV	Pt nanoparticles/poly(*o*-phenylenediamine)/GCE	0.7	2–10	*Melissa officinalis*	[175]
Protocatechuic Acid			0.7	1–35		
Chlorogenic acid	CV	Poly(aminosulfonic acid)/GCE	0.080	0.40–12	Traditional Chinese herbal medicines	[176]
	AdASWV	Highly defective mesoporous carbon–1-Butyl-3-methylimidazolium hexafluorophosphate paste electrode	0.01	0.02–2.5	*Echinacea purpurea, Calendula officinalis flowers extracts*	[177]
Ascorbic acid	AdCSWV ^10^	Mn(thiophen-2-carboxylic acid)_2_(triethanolamine) complex/Graphene oxide paste electrode	0.00697	0.0222–0.897	*Rosa canina* hips	[178]
	AdSDPV ^11^	Sepiolite clay nanoparticle–CPE	0.0042	0.014–0.9	Natural *Rosa canina* tea	[179]
	Potentiometry	Iodine-modified Pt electrode	—	10.0–1000	Aqueous extracts of rosehip fruits and hop cones	[180]
	DPV	GCE	5.05	20–1000	*Rosa* species of Turkey	[181]
Ascorbic acid	SWV	Hydroxyapatite-TiO_2_ composite/GCE	0.0633	2.78–2490	Clove oil and herbal decoction Kabasura Kudineer	[182]
Eugenol			0.094	1.4–78		
Eugenol	CV	GCE in 0.1 M Triton X100 medium	3.8	15–1230	Clove and sweet basil essential oils	[183]
	DPV	Graphene/GCE	0.007	0.10–17	Clove–lemon herbal pastille	[184]
	DPV	CeO_2_ nanoparticles dispersed in cetylpyridinium bromide/GCE	0.0191	0.075–75.0	Clove, cinnamon, basil, and nutmeg essential oils	[185]
	SWV	Immobilized eugenol–Carbon black nanoparticles in dihexadecyl hydrogen phosphate/GCE	0.00013 ^12^	0.029–26 ^12^	Clove oil	[186]
Anethole	SWV	La_2_O_3_ nanoparticles–CPE	—	9.45–28.3	Anise essential oil	[187]

^1^ Linear sweep voltammetry. ^2^ Graphite paste electrode. ^3^ Adsorptive anodic square-wave voltammetry. ^4^ Single-walled carbon nanotubes ^5^ Carbon fiber paper electrode. ^6^ Carbon paste electrode. ^7^ Molecularly imprinted polymer. ^8^ Adsorptive anodic differential pulse voltammetry. ^9^ Electrochemically activated screen-printed electrode. ^10^ Adsorptive cathodic square-wave voltammetry. ^11^ Adsorptive stripping differential pulse voltammetry. ^12^ µg.

**Figure 15:**
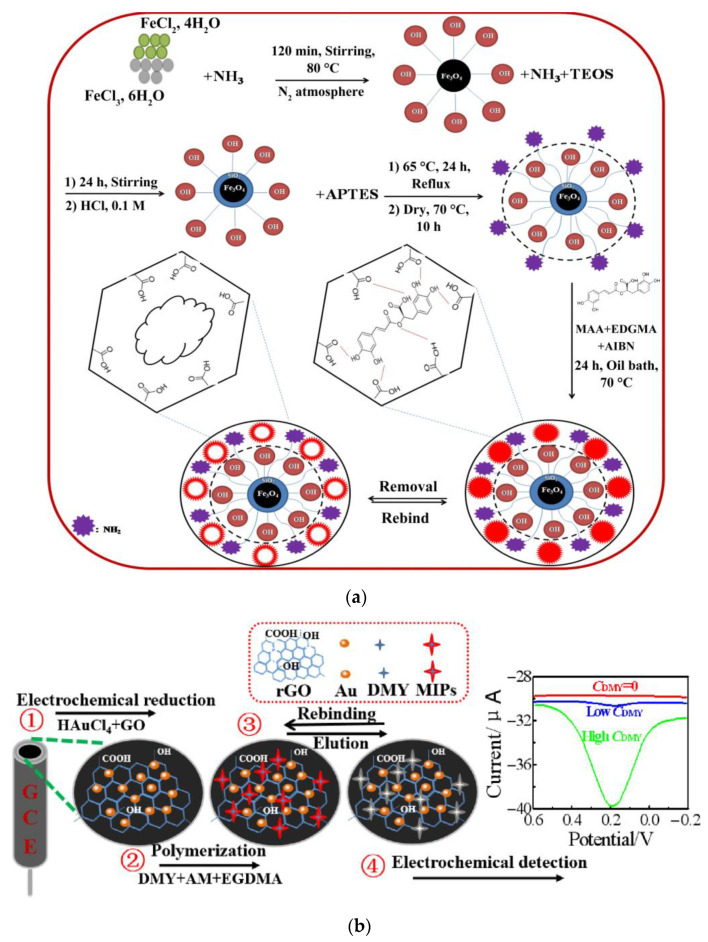
(**a**) Schematic representation of the preparation of rosmarinic acid molecularly imprinted polymer based on the magnetic Fe_3_O_4_@SiO_2_@NH_2_ decorated poly(methacrylic acid). Reprinted from [174], Copyright 2020, with permission of Elsevier. (**b**) Schematic view of the fabrication of dihydromyricetin (DMY) molecularly imprinted polymer (MIP) based on polyacrylamide with double-layered membranes consisting of gold nanoparticles (Au) anchored on reduced graphene oxide (rGO) on the GCE surface. Reprinted from [158]; published under the Creative Commons Attribution License.

Electrochemical synthesis of nanocoverages is favorable over chemical synthesis due to simplicity, high reproducibility of the final material, possibility to control thickness, and less tedious procedure (not more than 1 h instead of 3–24 h for chemical methods). Furthermore, chemical synthesis is usually multi-stepped (synthesis, cleaning, drying, and confirmation of the structure on each step) and requires the application of many reagents (some of them are toxic) and special equipment (Figure 15a). From this point of view, the electrochemical approach allows the obtaining of the final coverage via several or even one electrochemical step [147,153,158,169,173,175,176] (Figure 15b).

As one can see from Table 5, the linear response for nanomolar and lower concentrations has been achieved for morin [153,155] and hyperin [160,161,162] using an appropriate electrode surface modifier. Other antioxidants can be quantified at higher concentrations. However, the analytical characteristics obtained are sufficient for application to plant samples analysis. Regarding selectivity, the most impressive data were obtained using electrodes modified with molecularly imprinted polymers [158,172,174]. Although, other electrodes for flavonoids and phenolic acids determination [146,147,148,149,150,151,152,153,154,155,156,157,158,160,161,162,164,165,166,167,168,173,174,175,178] also show high selectivity in the presence of structurally related antioxidants that is caused by the type of modifier and conditions of electrochemical measurements (supporting electrolyte type and pH, electrochemical window, potential scan rate, etc.).

**Figure 16:**
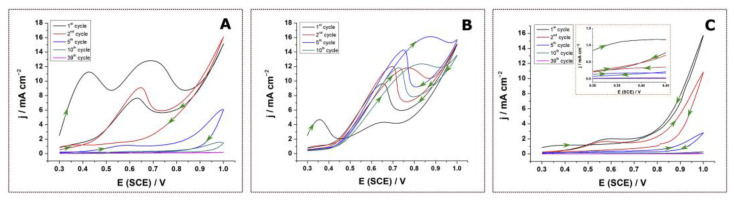
Voltamperometric cycles at 50 mVs^−1^ with Cu electrodes in (**A**) *O. vulgare*, (**B**) *O. x applii,* and (**C**) *T. vulgaris* essential oils in hydroalcoholic solution (inset corresponds to record detail in region between 0.30 and 0.45 V). Reprinted from [188], Copyright 2020, with permission from Elsevier.

An original approach for the selective detection of carvacrol in essential oils has been developed using the ability of carvacrol to be polymerized on the surface of copper electrodes, forming an insulating coverage [188]. Cyclic voltammetry or potentiostatic mode at 0.40 V can be successfully applied for the formation of a bright polymer. Thymol being a positional isomer of carvacrol does not electropolymerize. Therefore, there is no interference effect from thymol in carvacrol detection. The method is applicable for fast testing of carvacrol-containing essential oils (*Origanum vulgare*, *Origanum x applii*, *Thymus vulgaris*) (Figure 16). Two oxidation peaks are observed during the first cycle for *O. vulgare* (Figure 16A). One of them, close to 0.40 V, drastically decreases to zero during the second cycle. The second peak close to 0.70 V becomes lower but still significant during the second cycle. The voltammogram obtained with *O. x applii* (Figure 16B) during the first cycle shows a contribution in the vicinity of 0.40 V which disappears during the second cycle, as observed in the case of pure carvacrol and *O. vulgare*. There is another broad contribution at 0.60 V that may be associated with thymol and/or other components that are oxidized near the anodic limit of the potentiodynamic record. Additionally, the current density at the anodic limit does not markedly decrease upon cycling as in the case of pure carvacrol and thymol. The height of the peak at 0.40 V for the essential oils follows as *O. vulgare* > *O. x applii* > *T. vulgaris* revealing the same order of the content of carvacrol in the essential oils. In the case of *T. vulgaris* (Figure 16C), the current densities at 0.30–0.80 V are significantly lower than for other essential oils that agree well with a high level of thymol (78.4%) and low contents of carvacrol (2%). Although the signal at 0.40 V is low, it is near zero during the second cycle. Notwithstanding the concentration of thymol is high in this essential oil, its oxidation peak is low and seems to be hindered by the other components of the essential oil.

Therefore, considering the results of Figure 16, the presence of carvacrol in the essential oils can be identified by analyzing comparatively the first and the second potentiodynamic cycles: if the current density recorded in the vicinity of 0.40 V in the first cycle dropped to zero during the second one, this contribution could be attributed to carvacrol. Unfortunately, the quantification of carvacrol is impossible. Moreover, essential oils with other major phenolic terpenes containing trace carvacrol cannot be studied as far as the major component will also undergo electropolymerization [189,190,191].

Individual voltammetric quantification of isopropylmethylphenols (thymol and carvacrol) can be realized using chemometric treatment of electrochemical data. Successful selective determination of carvacrol in oregano essential oil has been achieved by a novel method using voltammetric second-order modeling based on multivariate curve resolution-alternating least-square [192]. Second-order cyclic voltammetry data have been generated using various potential scan rates. Differentiation of carvacrol and thymol has been performed. The possibility of simultaneous differential pulse voltammetric determination of eugenol, carvacrol, and thymol on the boron-doped diamond electrode has been shown using a multivariate partial least-squares calibration model [193]. Low complexity and simple data pre-processing procedures have been chosen for partial least-squares model optimization. The data obtained allow reliable identification and quantification of analytes, as well as rapid, accurate, and low-cost verification of the authenticity of commercial products (herbs and essential oils).

## 6. Conclusions and Future Development

Electrochemical methods are a promising tool for the characterization of antioxidant properties of medicinal plants and products of their basis. The simplicity of equipment, rapidity of analytical procedures, low reagent consumption, reliable data, and good metrological and operation characteristics are important advantages over traditional methods such as spectroscopy and chromatography. Electrochemical methods have no limitations in the analysis of a colored sample that is typical for medicinal plants and their extracts. The use of oxidants as reagents or own oxidation of antioxidants at the electrode surface excludes the application of radical species such as DPPH^•^ sensitive to light and water contents in solution. Furthermore, the modern trend in miniaturization of electrochemical equipment and the appearance of portable electrochemical analyzers operated by smartphones allow their application outside the laboratories that are useful for the operative control of plant material in place of their growth or storage.

Current developments in electroanalysis allow the measurement of total antioxidant parameters characterizing medicinal plant-based samples as a whole, taking into account possible synergetic and antagonistic effects. This is useful for the characterization and prediction of the biological activity of the samples. Voltammetric determination of specific classes of antioxidants (phenolic acids, flavonoids, capsaicinoids, isopropylmethylphenols) can be used for the characterization of medicinal plants depending on the stage, place, and conditions of growth, chemotype elucidation, as well as for the choice of most perspective plants for the isolation of these antioxidants and following the production of pharmaceutical on their basis. Selective electrochemical determination of individual antioxidants is a more complex task and can be solved by the application of chemically modified electrodes.

Voltammetry is the most used electrochemical method due to the possibility to measure both the total and individual contents of antioxidants in medicinal plants. The possibility of electrode surface modification opens wide opportunities for the sensitivity and selectivity control of the antioxidant response in a complex matrix. On the other hand, constant-current coulometry and potentiometry are preferable for the total antioxidant parameters evaluation due to the wide range of antioxidants to be measured, simplicity of the protocols, high reproducibility of the data obtained, and rapidity in comparison to methods based on the electrooxidation of antioxidants on the electrode surface. Moreover, coulometry and potentiometry can be used for routine practice by specialists in pharmacognosy, pharmacy, biology, biochemistry, etc., as far as these methods do not require high skills in electrochemistry.

Further developments in the electrochemical evaluation of medicinal plants can be focused on the following aspects:

- To date, extracts with toxic solvents have often been studied to characterize the antioxidant constituents of the medicinal plant. Investigation of the medicinal plants’ dosage forms such as infusions, decoctions, tinctures, and essential oils has to be enlarged. Essential oils are almost out of the investigation, although of high interest;
- Unification of standard antioxidants used to express the total antioxidant parameters.
- Development of electrochemical methods for the determination of tocopherols and terpenes in medicinal plants and products on their basis. Total contents of tocopherols and tocotrienols or terpenes and their individual quantification are required;
- Enlargement of individual antioxidants studied. A limited range of natural phenolics are considered to date, whereas many other important for medicinal plants antioxidants and even classes of antioxidants are out of investigation;
- Application of flow systems including microfluidic chips that can significantly improve the operating characteristics of the systems and decrease electrode fouling and interference effects of co-existing components;
- Attention should be paid to the methods of electrochemical generation of reactive oxygen species and detection of their reaction to antioxidants;
- Creation of novel highly selective electrodes for the simultaneous determination of the antioxidants of the same class or group including isomers for the phytochemical profiling of the plant samples. Novel functional materials acting as electrode surface modifiers are required and can be predicted using molecular modeling and quantum chemical calculations with the following controlled synthesis.

In general, electrochemical methods are a good alternative to spectroscopy and chromatography. They have an excellent perspective in the characterization of the antioxidant properties of medicinal plants and products on their basis. The practical application of electrochemical protocols has to be expanded.

## Figures and Tables

**Table 1:** Phenolic antioxidants of medicinal plants.

Medicinal Plant	Phenolic Antioxidants	Refs.
*Aloe vera *(L.) Webb.	Flavonoids (quercetin, kaempferol, apigenin, catechins, rutin, myricetin), hydroxybenzoic (gallic, protocatechuic, vanillic, syringic, gentisic), and hydroxycinnamic (sinapic, chlorogenic, caffeic, coumaric, ferulic) acids	[48]
*Aralia elata* (Miq.) Seem.	Phenolic acids (gallic, ferulic, sinapic, caffeic, chlorogenic, 3,5-di-O-caffeoylquinic, cryptochlorogenic, neochlorogenic, protocatechuic), flavonoids (catechin, epicatechin, quercetin, kaempferol, and their glycosides), coumarins, and lignans	[49,50]
*Calendula officinalis* L.	Flavonoids (quercetin, isorhamnetin, and their glycosides, isoquercetin, narcissin, calendoflaside, calendoflavoside, calendoflavobioside, neohesperidoside)	[51,52]
*Capsicum annuum* L.	Capsaicinoids, hydroxybenzoic and hydroxycinnamic acids; flavonoids (quercetin, kaempferol, apigenin, luteolin, and their glycosides, catechins), resveratrol, anthocyanidins, and coumarins	[53]
*Crataegus* spp.	Procyanidins (epicatechin-type oligomers), flavonoids (vitexin, quercetin, hyperoside, luteolin, and their glycosides, rutin), chlorogenic acid	[54]
*Echinacea purpurea* (L.) Moench	Caffeic acid and its derivatives (caftaric acid, chlorogenic acid, cynarin, echinacoside, and cichoric acid), rutin	[55,56]
*Eleutherococcus senticosus* (Rupr. & Maxim.) Maxim.	Lignans (eleutherosides), flavonoids (rutoside, hyperoside, prunin, isoquercetin, kaempferol glycosides), hydroxycinnamic (chlorogenic, ferulic, rosmarinic, caffeic), and hydroxybenzoic (protocatechuic, vanillic, gallic) acids	[57,58,59,60]
*Eucalyptus* spp.	Flavonoids (quercetin, rutin, catechin) and phenolic acids (gallic, chlorogenic, and ellagic)	[61]
*Hypericum perforatum* L.	Hydroxycinnamic acids (chlorogenic, cryptochlorogenic, neochlorogenic, ferulic, caffeic, 4- and 5-O-*p*-coumaroylquinic acids), protocatechuic acid, flavonoids (hyperoside, isoquercitrin, miquelianin, quercitrin, quercetin, rutin, hyperforin, adhyperforin, hypericin, biapigenin)	[62,63]
*Ginkgo biloba* L.	Flavonoids (quercetin, kaempferol, isorhamnetin, luteolin, and their glycosides, myricetin, morin), hydroxybenzoic (protocatechuic, syringic) and hydroxycinnamic (chlorogenic, caffeic, *p*-coumaric, rosmarinic) acids	[64,65]
*Leonurus cardiaca* L.	Hydroxybenzoic (*p*-hydroxybenzoic, vanillic) and hydroxycinnamic (chlorogenic, caffeic, caffeoylmalic, and *trans*-ferulic) acids, flavonoids (rutin and quercetin), phenylethanoid glycosides (verbascoside and lavandulifolioside)	[66]
*Matricaria chamomilla* L.	Flavonoids (apigenin, luteolin and their 7-O-glucosides, quercetin, rutin) and hydroxycinnamic (chlorogenic, caffeic, ferulic, *p*-coumaric) acids	[67]
*Mentha x piperita* L.	Flavonoids (eriodictyol, luteolin, hesperetin, and apigenin glycosides, pebrellin, and gardenin B), hydroxycinnamic acids (rosmarinic and caffeic)	[68]
*Ocimum sanctum* L.	Flavonoids (apigenin, vitexin, isovitexin, vicenin 2, apigenin-7-*O*-glucuronide, luteolin, orientin, isorientin, galuteolin, luteolin-7-*O*-glucuronide, quercetin), aesculin, hydroxycinnamic acids (caffeic, rosmarinic, chlorogenic)	[69]
*Paeonia anomala* L.	Flavonoids (onopordin and its derivatives, quercetin glycosides), tannins, fischeroside B, gnetin H, resveratrol, ellagic acid, ethyl- and methylgallates	[70,71]
*Panax ginseng* C. A. Meyer	Hydroxybenzoic (*p*-hydroxybenzoic, vanillic, protocatechuic, syringic, gentisic) and hydroxycinnamic (caffeic, *p*-, *o*-and *m*-coumaric and ferulic) acids, flavonoids (rutin, catechin, epicatechin, epigallocatechin gallate, quercitrin, apigenin, myricetin, morin, quercetin, kaempferol, naringenin, naringin), resveratrol, gomisins A and N^51^	[72,73]
*Rhodiola rosea* L.	Salidroside and its aglycon tyrosol, rosavin, rosarin, rosin, flavonoids (catechin, epigallocatechin, epigallocatechin gallate, quercetin, rutin), hydroxybenzoic (gallic, protocatechuic, vanillic) and hydroxycinnamic (caffeic, *p*-coumaric, ferulic, sinapic) acids	[74,75,76]
*Silybum marianum* (L.) Gaertn.	Hydroxycinnamic acids (caffeic, ferulic, chlorogenic), flavonoids (luteolin, myricetin, apigenin, silybin)	[77]
*Schisandra chinensis* (Turcz.) Baill.	Dibenzocyclooctadiene lignans (schisandrins A, B, C, gomisins, schisantherins and etc.), hydroxycinnamic (chlorogenic, *p*-coumaric) and hydroxybenzoic (*p*-hydroxybenzoic, protocatechuic, syringic and trace amounts of gentisic) acids, flavonoids (hyperoside, rutin, quercetin, and isoquercitrin)	[78]
*Syzygium aromaticum* L.	Eugenol, vanillin, carvacrol, flavonoids (kaempferol, rhamnetin, myricetin, quercetin), eugenitin and eugenin, biflorin, ellagic, and gallic acids	[79]
*Thymus vulgaris* L.	Thymol, carvacrol, flavonoids (6-hydroxyluteolin, apigenin, luteolin, cirsimaritin or genkwanin, cirsilineol, 5-desmethylnobiletin, 8-methoxycirsilineol, 7-methoxyluteolin, gardenin B, salvigenin, thymonin, sideritoflavone, xanthomicrol, thymusin), hydroxycinnamic (rosmarinic, caffeic, *p*-coumaric, ferulic) and hydroxybenzoic (*p*-hydroxybenzoic, gentisic, syringic) acids	[80]
*Valeriana officinalis* L.	Hydroxycinnamic acids (caffeic, chlorogenic, and isoferulic), lignans ((+)-hydroxypinoresinol and pinoresinol), flavonoids (luteolin, diosmetin, kaempferol, apigenin, linarin)	[81]

**Table 2:** Voltammetric evaluation of superoxide scavenging capacity of medicinal plants.

Electrode	Supporting Electrolyte	Superoxide Scavenging Capacity Expression	Plant Samples	Refs.
GCE	0.5 M Bu_4_NPF_6_ in DMF	*I*_a50_	*Ascophyllum nodosum* phenol fractions	[97]
	0.1 M Bu_4_NPF_6_ in DMF	*I*_a_ (%) in ascorbic acid equivalents	*Limoniastrum feei* aqueous extract	[98]
	0.1 M Bu_4_NBF_4_ in DMF	*I*_a50_	*Cupressus sempervirens* L. extracts	[99]
	0.1 M Bu_4_NClO_4_ in DMSO	Antioxidant coefficient (K_ao_)	*Berberis lycium* Royel, *Zanthoxylum armatum*, and *Morus nigra* L.	[100]
	0.1 M Bu_4_NClO_4_ in acetonitrile	Antioxidant coefficient (K_ao_)	*Ixora coccinea* Linn. root extract	[101]
Pt	0.1 M Et_4_NCl in ethanol	Antioxidant coefficient (K_ao_)	5 *Mentha* species essential oils	[102]
MWCNTs/GCE	0.05 M Et_4_NI in DMF	*I*_c_ in gallic acid equivalents	11 medicinal herb tinctures	[103]

**Table 3:** Analytical capabilities of pulse voltammetric modes in characterization of medicinal plants antioxidant properties.

Method	Electrode	Supporting Electrolyte	Antioxidant Parameters	Plant Samples	Refs.
DPV	Pt	0.1 M Bu_4_NClO_4_ in acetonitrile	EI	Ethanolic extracts of Douglas fir, Scots pine, and Korean fir cones	[117]
	GCE	0.1 M NaClO_4_ in methanol	Total electrochemical antioxidant power in ascorbic acid equivalents	*Methanolic extracts from Cytisus multiflorus*, *Filipendula ulmaria,* and *Sambucus nigra* flowers	[120]
		0.1 M NaClO_4_ and 1 mM camphorsulfonic acid in 0.2 M PB ^1^ pH 7.4.	Total antioxidant potential (integral under the DPV curve)	14 herbal extracts	[121]
		0.1 M PB pH 6.0	EI	10 herbal ethanolic extracts	[122]
	CNTs in 0.5% CS ^2^/GCE	Britton-Robinson buffer pH 3.0	Total content of polyphenols Antioxidant capacity equivalent to ascorbic or gallic acids	*Echinacea purpurea*-based capsules, tablets, tincture	[123]
SWV	GCE	Britton-Robinson buffer pH 1.8	Total polyphenol contents (area under the curve)	Roots of three *Echinacea* *purpurea* species	[124]
		Britton-Robinson buffer pH 2.5	TAC in gallic acid equivalents (area under the curve)	CO_2_ supercritical fluid extracts from *Matricaria chamomilla*, *Achillea millefolium*, *Helichrysum italicum,* and *Hypericum perforatum*	[125]

^1^ Phosphate buffer. ^2^ Carbon nanotubes dispersed in 0.5% chitosan.

**Table 4:** Advantages and disadvantages of the electrochemical methods used for the evaluation of total antioxidant parameters of medicinal plants.

Method	Advantages	Disadvantages
Constant-current coulometry	Method is universal, simple, does not require standard antioxidants, is highly sensitive, and rapid. Low consumption of sample and possibility of automation, low cost.	Possible impact of non-antioxidants due to the relatively high reactivity of the electrogenerated bromine. Basic medium for the generation of ferrocyanide ions can cause chemical oxidation of the antioxidants with air oxygen.
Potentiometry	Simplicity, low cost, the possibility of miniaturization and automation.	Necessity to use calibration. Estimation of high concentrations of the antioxidants due to low sensitivity. Limited range of antioxidants.
Voltammetry using DPPH^•^	Rapidity, automation, and high throughput in flow mode. DPPH^•^ immobilization on the electrode surface simplifies the procedure and reduces reagent consumption.	Limited range of reactive antioxidants. Insufficient stability of the reagent in solution and its applicability in organic medium and mainly to the liposoluble antioxidants. Low similarity of DPPH^•^ to radicals acting in living systems.
Voltammetry using O_2_^•–^	Rapidity, simplicity, and no need to use additional reagents. O_2_^•–^ is a reactive oxygen specimen that occurs in living systems.	Necessity to use organic solvents and inapplicability in water media. Limited range of antioxidants to be studied. Narrow range of antioxidant concentrations due to the distortion of voltammograms shape making impossible calculations.
Voltammetry using DNA-modified electrodes	Application of Fenton reaction occurred in biological systems. Possibility of device miniaturization.	DNA-immobilization technology, stability of the DNA-modified electrode. Effect of ^•^OH on DNA in the cell and on the electrode is different. Fenton reactive mixture components and components of the sample can also affect the DNA. Sensitivity can be improved. Relatively high cost.
Amperometric flow-injection analysis with copper(II)-neocuproine complex	Simplicity, rapidity, automation, high throughput, selectivity, low-cost, portability, and reagent stability.	Necessity to use standard antioxidant, chelating, or complexing compounds in the sample can affect the measurement results.
Voltammetric EI	Method is universal, simple, rapid, low cost, and does not require additional reagents.	Only major components with high concentrations are measured. Impact of ascorbic acid or tocopherols cannot be excluded.
Chrono methods	Method is universal, simple, rapid, low cost, and does not require additional reagents. Possibility of device miniaturization and operated using smartphone. Contribution of all components is taken into account.	Necessity of the electrode surface modification for sufficient sensitivity. Application of a standard antioxidant for comparison to other methods.

## Data Availability

Not applicable.
